# Hesperidin improves insulin resistance via down-regulation of inflammatory responses: Biochemical analysis and *in silico* validation

**DOI:** 10.1371/journal.pone.0227637

**Published:** 2020-01-13

**Authors:** Kanwal Rehman, Syeda Mehak Munawar, Muhammad Sajid Hamid Akash, Manal Ali Buabeid, Tahir Ali Chohan, Muhammad Tariq, Komal Jabeen, El-Shaimaa A. Arafa

**Affiliations:** 1 Department of Pharmacy, University of Agriculture, Faisalabad, Pakistan; 2 Institute of Physiology and Pharmacology, University of Agriculture, Faisalabad, Pakistan; 3 Department of Pharmaceutical Chemistry, Government College University, Faisalabad, Pakistan; 4 Department of Clinical Sciences, College of Pharmacy and Health Sciences, Ajman University, Ajman, United Arab Emirates; 5 Institute of Pharmaceutical Sciences, University of Veterinary and Animal Sciences, Lahore, Pakistan; 6 Department of Pharmacology, Lahore Pharmacy College, Lahore, Pakistan; Dasman Diabetes Institute, KUWAIT

## Abstract

Leptin resistance and co-existing insulin resistance is considered as hallmark of diet-induced obesity. Here, we investigated therapeutic potential of hesperidin to improve leptin and insulin resistance using high fat diet (HFD)-induced obese experimental animal model. We also performed *in silico* studies to validate therapeutic effectiveness of hesperidin by performing protein-ligand docking and molecular dynamics simulation studies. Group 1 was identified as control group receiving vehicle only. Group 2 was marked as non-treated group receiving 60% HFD. While, other groups were treated daily with orlistat (120 mg/kg/d), hesperidin (55 mg/kg/d), combination of hesperidin (55 mg/kg/d) + orlistat (120 mg/kg/d). Hesperidin alone (P<0.001) and particularly in combination with orlistat (P<0.001), resulted in controlling the levels of HFD-altered biomarkers including random and fasting state of glycemia, leptin and insulin resistance. Similarly, hesperidin also improved the serum and tissue levels of leptin, interleukin-6 and tumor necrosis factor-alpha more significantly (P<0.05) when compared with that of orlistat. These results were found to be in accordance with the results of histopathological examination of pancreas, liver and adipose tissues. *In-silico* studies also proved that hesperidin binds to leptin receptor with higher affinity as compared to that of orlistat and induces the favorable variations in geometrical conformation of leptin receptor to promote its association with leptin which may lead to the cascades of reactions culminating the lipolysis of fats that may ultimately lead to cure obesity. The results of this study may be a significant expectation among the forthcoming treatment strategies for leptin and insulin resistance.

## Introduction

Leptin, a hormonal peptide is known to control the body weight. Leptin is produced primarily by white adipose tissues. Other factors that may contribute in increasing the secretion of leptin includes reduce food intake and/or excess utilization of body energy through hypothalamic-pituitary-gonadal axis [1]. However, leptin is an adipocyte-derived hormone. Moreover, the amount of fat tissues are known to have influence on the concentration of leptin in systemic circulation [2]. Any change in the level of leptin secretion may have a direct influence on metabolic functions of the body. This may be because of the contribution of leptin for oxidation of free fatty acids (FFAs) in periphery, which in turn results in the decreased accumulation of body fat. Similarly, leptin also plays its role centrally through hypothalamus for regulating the food intake. In addition, even in the presence of hyperleptinemia, insulin resistance, a hallmark of diabetes mellitus (DM), may also contribute in loss of leptin sensitivity particularly in certain conditions like obesity that may lead to leptin resistance [3, 4]. Hyperleptinemia and leptin resistance in turn may cause disturbances in lipid metabolism causing reduction in FFAs oxidation and increasing the levels of triglycerides (TGs) [5]. However, makeover of leptin sensitivity has been suggested to be helpful in ameliorating the disturbances in lipid profile and associated conditions like DM [6–8].

Up till now, different agents have been approved as an anti-obesity drugs for the treatment and/or management of obesity. Orlistat is also an anti-obesity drug used commercially available for the treatment of abnormalities in lipid profile. It is a useful drug which has been reported to promote weight loss by decreasing the serum level of leptin and insulin [9, 10]. Orlistat has been well recognized as a reversible inhibitor of pancreatic and gastric lipases. Moreover, it has also shown to decrease the absorption of dietary fat. It has also shown its effects against hypertension and DM [11, 12]. However, the use of orlistat may be underlying the risk of sub-acute liver toxicity and steatorrhea. Although, a very few cases have been reported for clinical sign of acute liver injury caused by orlistat, in 2010, food and drug administration (FDA) proclaimed safety concerns regarding orlistat-induced liver injury. Among reported cases, the onset of injury was observed to be between 2 to 12 weeks of starting orlistat [13]. Similarly, it has been suggested after evaluating the effects of orlistat on live that idiosyncratic liver damage related to orlistat cannot be neglected, it is likely to be extremely rare. Long-term use of orlistat may cause liver injury. Patients should be carefully monitored for the signs of liver dysfunction caused by orlistat. Moreover, there is no known therapy for the orlistat-induced liver toxicity yet [14].

More than 5000 of phenolic compounds have been identified, where the major contribution for therapeutic activities belongs to the class of flavonoids that are known to be present in various vegetables and fruits [10]. Hesperidin present in many citrus fruits, has been recognized in recent years as a potential flavonoid for therapeutic effects [15]. Hesperidin has shown potent antioxidant ability in many experimental studies [16, 17]. Hesperidin being an antioxidant, has also shown some other positive effects through down-regulation of cytokines including tumor necrosis factor-alpha (TNF-α) and interleukin-1 beta (IL-1β) contributing for its anti-inflammatory effects [18]. In addition, hesperidin manages to regulate glucolipotoxicity and reduces neuropathic pain during DM. Similarly, on comparative analysis of hesperidin with other synthetic and/or naturally available antioxidants like butylated hydroxyanisole and hydroxytoluene (BHA and BHT), α- tocopherol and vitamin-C, it showed better scavenging effects on free radicals of H^+^ and H_2_O_2_ [19–23]. Moreover, synergistic efficacy of hesperidin with other citrus flavonoids for instance lemon flavonoids, like eriocitrin has also demonstrated admirable potential to reduce oxidative stress in diabetic rats [24, 25]. Other therapeutic effects of hesperidin that has been revealed in recent years, include the regulation of lipid and glucose metabolizing enzymes [26] and refining cardiac functions [27]. However, to date, no reports have focused on the effect of hesperidin on obese gene product i.e. leptin in comparison to already approved anti-obesity drug in hyperlipidemic and hyperglycemic conditions. Hence, in the current work, we evaluated the hypolipidemic and anti-obesity effects of hesperidin in relation to its effects on leptin in serum and adipose tissues. Moreover, protective effect of hesperidin treatment on liver as compared to that of the orlistat has also been observed. In addition, *in silico* studies using computational docking and molecular dynamics simulation tools were also performed for the verification and validation of *in vivo* study conducted on albino rats as experimentally high fat diet (HFD)-induced obesity model proclaiming the strength and binding affinity of orlistat and hesperidin with leptin or its receptors.

## Materials and methods

### Chemicals

HFD (60%) was prepared according to its protocol (Open Source Diets^®^). Orlistat (Xenical^®^) was purchased from local pharmacy, while hesperidin and all other chemicals used were of analytical grade.

### Experimentally HFD-induced obesity animal model

About 30 weaned/adult Wistar albino rats weighing around 145–200 g, were locally purchased and kept at animal house of University of Agriculture Faisalabad (UAF), Pakistan at ambient temperature (25 ± 5°C). Before the start of experiments, rats were fed on normal diet with water *ad libitum* allowed to acclimatize for two weeks. All experimental procedures were carried out in accordance with the approved laboratory animal biosafety guidelines and protocol of Institutional Biosafety committee (IBC) of UAF (No. DGS/2785-88). The body weight of all experimental groups was recorded before and on weekly basis throughout the study period. After a week of acclimation, the rats were divided into 5 groups. Group 1 was named as **NC** group that was receiving normal diet with water *ad libitum* throughout the study period. Group 2 was designated as **HFD** group to which HFD was given without any other treatment. Other three groups were also fed with HFD however, they were latter provided the treatment and were divided as follows; Group 3 was called as **ORL** and received 120 mg/kg/d p.o. Group 4 was marked as **HES** and received 55 mg/kg/d p.o. While, the last group treated with combination of orlistat (120 mg/kg/d) p.o. and hesperidin (55 mg/kg/d) p.o. was marked as **ORL + HES** group.

### Biochemical analysis

About 1 ml blood sample was collected by tail vein method from each rat of every group before the start of treatment, at 15^th^ (during) and 30^th^ day (end) of treatment period. Blood samples were centrifuged for approximately 15 min at 3000 × g and the serum were separated and stored at freezing temperature (-20 ^o^C) till the further analysis of biochemical parameters.

### Assessment of glycemic control biomarkers

Fasting and random blood glucose levels for each group were measured with help of glucometer 2 times a week for the estimation of effect of given treatment on glycemic levels of animals. The serum level of insulin was estimated before, during and at the end of treatment period using assay kit. Before the end of treatment, oral glucose tolerance test (OGTT) was performed to evaluate the tendency of glucose tolerance in experimentally-induced obese animals. Before performing the protocol of OGTT, rats were fasted overnight. The blood samples were collected at predefined time points. Fasting blood glucose level of each group was measured with the help of glucometer. Followed by the administration of glucose (2 gm/kg) solution by oral gavage, blood glucose levels were measured at 30, 60, 90 and 120 min. Before the administration of glucose, we also measured the fasting level of serum insulin to assess the effect of treatment on insulin resistance with the help of HOMA-IR (homeostatic model assessment of insulin resistance).

### Assessment of lipid profile biomarkers

To assess the impact of treatment on lipid profile, we measured the serum levels of cholesterol, triglycerides (TGs), high density lipoproteins (HDL) and low-density lipoprotein (LDL) using their corresponding assay reagent kits.

### Assessment of leptin and pro-inflammatory biomarkers

Leptin, the peptide hormone, exhibits an important role in adiposity and appetite. Leptin was estimated in serum and tissue homogenates of adipose tissues by ELISA detection method using its corresponding assay kit. Cytokines including interleukin-6 (IL-6) and TNF-α were measured in the serum and tissue homogenates of the treated and non-treated animals by ELISA detection method using their corresponding assay kits.

### Assessment of liver and kidney function biomarkers

Aspartate aminotransferase (AST), alanine aminotransferase (ALT), blood urea nitrogen (BUN) and creatinine are well recognized to depict the normal function of liver and kidney. In current study, to assess the effect of treatment on liver and kidney function, the serum levels of AST, ALT, BUN and creatinine were measured before, during and at the end of treatment period using their commercially available assay kits.

### Sampling of tissues for protein expression and histopathological examination

At the end of treatment, rats were sacrificed by cervical dislocation and the abdomen was dissected, pancreas, kidney and liver were removed for histopathological analysis. To estimate the impact of treatment on the leptin, IL-6 and TNF-α content in tissue, adipose tissue was collected and stored at 10 ^o^C in dark place. For the process of homogenization, firstly, the lysis buffer was prepared to help deteriorate fatty and nuclei membranes surrounding the cells and within the cells. About 0.1 M phosphate buffered saline (PBS) was used for the homogenization of white adipose tissue. Tissue sample was then taken in falcon tube already having 0.1 M PBS. The tube was placed under tissue homogenizer for homogenization at 3000 rpm. Homogenized tissue was stored in freezer at -20 ^o^C till the analysis of leptin content. Moreover, for histopathological analysis, tissues from the liver, pancreas and adipose were also collected and followed the procedure of fixation, tissue embedding, sectioning, mounting and staining according to the protocol for the histopathological analysis of concerned tissues of treated and non-treated animals of this study.

### Computational protein-ligand docking and molecular dynamics simulation studies

#### Molecular modeling and protein structures preparation

The FASTA sequences with NCBI Accession number P48357 and P41159 for leptin binding domain (LBD) of leptin receptor and leptin protein, respectively, were retrieved from UniProt [28, 29], and submitted to the I-TASSER online server [28]. Only the top-ranking I-TASSER-generated and -optimized 3D-conformations of both proteins according to the confidence score (C-score) were downloaded for molecular docking and molecular dynamics (MD) simulations. Prior to molecular docking and MD simulation studies, the generated models were energy-minimized and MD-simulated for 10 ns, which have been discussed in the later section.

#### Protein-protein docking

The complete and energy optimized structures of LBD and leptin protein were used to perform protein-protein docking using HEDDOCK online server [30] to investigate their lateral binding affinity. Easy interface module of HADDOCK webserver was selected to upload LBD and leptin protein as molecule-1 and molecule-2, respectively. The active sites residues for LBD (Phe504_Leu507) and leptin protein (Arg20 and Gln75) critically involved in lateral interactions [31, 32] were specified for molecule-1 and molecule-2, respectively.

#### Ligand preparation and docking studies

3D structures of orlistat (ORL) and hesperidin (HES) were constructed using SKETCH module implemented in Sybyl‐X1.3 [33]. The energies of selected inhibitors were minimized by applying Tripos force field along with GasteigereHückel atomic charge [34]. The constructed 3D-structures were further optimized with MD approach to attain active geometrical conformation. Flexible docking simulations were performed to reveal the binding modes of selected inhibitors in LBD-leptin complex using a Surflex‐Dock module of SYBYL‐X 1.3 [35]. At first, the structure of LBD-leptin complex was carefully analyzed to avoid any chemical inaccuracy, hydrogens were added, charges and atom types were assigned according to the AMBER 7 FF99 force field by adopting structure preparation tools applicable in the biopolymer module of SYBYL-X 1.3 [33]. Finally, energy was minimized for 1000 cycles by applying Powell algorithm along with convergence gradient of 0.5 kcal/(mol_Å). Surflex-docking utilizes ProtoMol [36], which mimic target site to generate putative poses of small molecules. The parameters to generate ProtoMol were kept at default values (threshold = 0.50 and bloat = 0). Finally, the energy-optimized active conformations of ORL and HES were individually docked into the LBD-leptin complex. Twenty best docked poses were saved conclusively for each inhibitor in its respective ligand–protein complex system. The putative poses of ligands were ranked according to Hammerhead scoring function (C-score) [33].

#### Molecular dynamics simulation studies

The top ranked docking-predicted conformers (LBD-leptin, ORL- and HES-LBD-leptin) were subjected to MD simulations using AMBER16 software package [37]. All three complex systems were neutralized by adding Na^+^ counter-ions. Each complex was immersed into an octahedron box of the TIP3P [38] water model of 12 Å dimension at 300 K temperature and 1 bar pressure. Each system of the protein-protein and protein-ligand complex was then subjected to production simulations run of 50 ns following the same protocol and parameters as those reported in our previous publications [39, 40]. All MD simulations were carried out using the CARNAL, ANAL, and PTRAJ modules of AMBER16.

#### Free energy calculation and decomposition analyses

Molecular mechanics-based scoring method MM/PB(GB)SA [41], was applied to compare the binding free energies of ORL and HES in ORL-LBD-leptin and HES-LBD-leptin complexes. In addition, the same methodology was applied to reveal binding affinities of LBD and leptin in LBD-leptin, ORL-LBD-leptin and HES-LBD-leptin complexes. The pairwise nature of GB methodology provides an opportunity to decompose free-energies into insightful interaction and desolation components. Hence, the protein-protein and ligand-protein interactions were further decomposed into per residue energy components. All MM/PB(GB)SA calculations [41] and per-residue free energy decomposition analyses were entirely performed in the AMBER16 software package, following the same protocol and parameters as those reported in our previous publications [39].

## Results

### Assessment of treatment on body weight and glycemic control biomarkers

**Fig 1:**
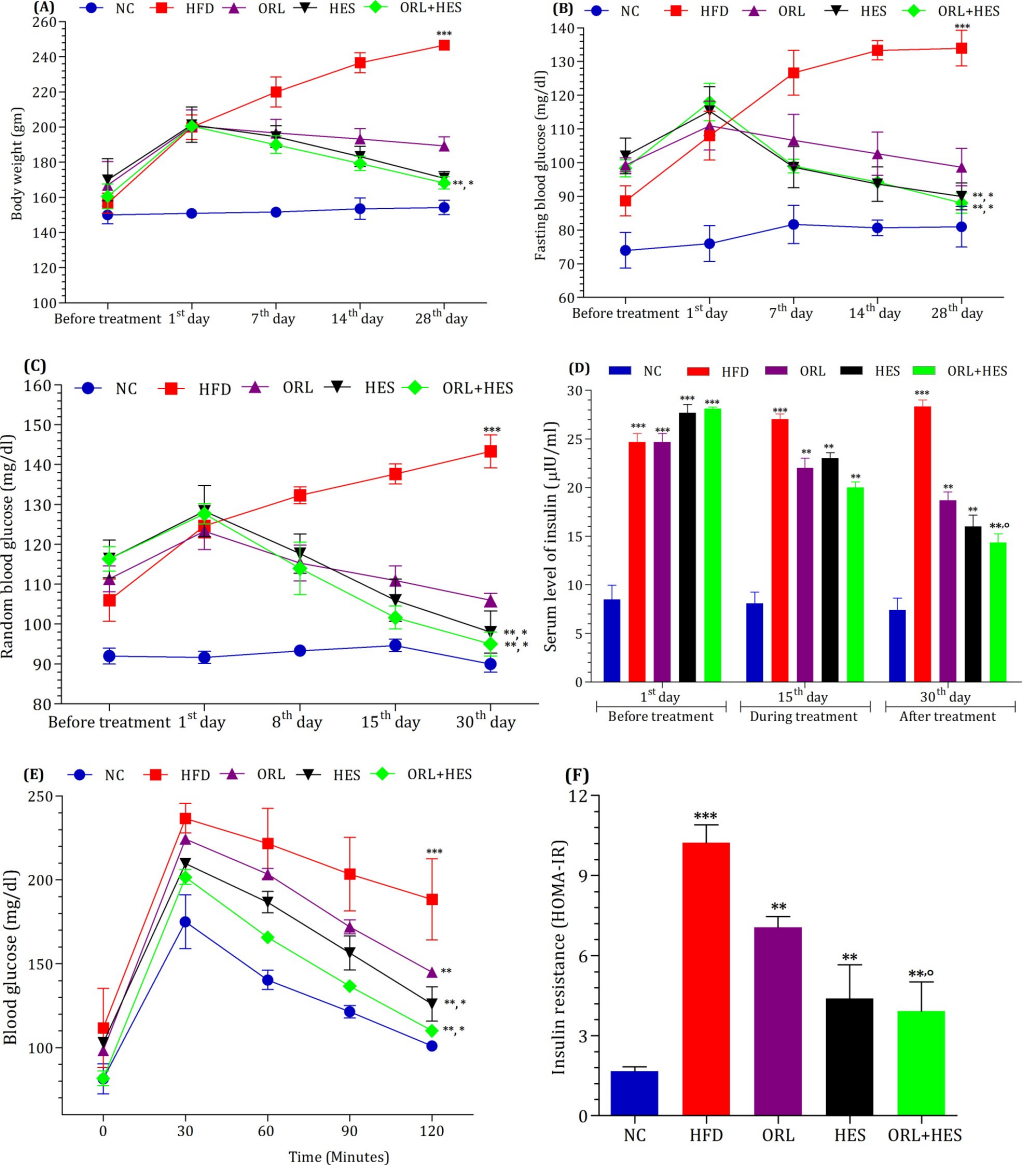
**Effect of treatment on (A) body weight and (B-F) glycemia**. The level of (B) fasting blood glucose and (C) random blood glucose was measured in all experimental groups on weekly basis. (D) Serum level of insulin from all experimental groups was measured at 1^st^, 15^th^, and 30^th^ day of the treatment period. Before the end of treatment, (E) oral glucose tolerance test (OGTT) was performed by administering glucose (2 mg/kg body weight of rat) after an overnight starvation and blood was collected at predefined time points. (F) Insulin resistance was calculated by HOMA-IR using the fasting levels of serum insulin and blood glucose. The level of significant difference was estimated using Bonferroni post-test having two-way ANOVA. *** represent P<0.001 when compared with control group. * represent P<0.001 when compared with the HFD group, ** represent P<0.001 when compared ORL, HES and ORL+HES groups with HFD group, ° represent P<0.05 when compared with ORL group at 30^th^ day of treatment.

To investigate the effect of treatment on body weight, experimental animals were weighed on weekly basis. HFD significantly elevated the body weight (*P*<0.001) as seen in HFD, ORL, HES and ORL+HES groups when compared with NC-group before the start of treatment (Fig 1A). However, it was observed that after administering the ORL, HES and/or the combination of ORL+HES to the designated groups of rats, the body weight of HES, ORL and ORL+HES groups was started to decrease (*P*<0.05) when compared with that of HFD-group. At the end of treatment, combination of ORL and HES significantly decreased the body weight (P<0.001) when compared with that of ORL alone. Though treatment with HES alone and with ORL reduced the body weight, however it slightly reduced the food intake also.

To estimate the effect of treatment on glycemia, we measured FBG and RBG before, during and at the end of treatment. We found that before the start of treatment, HFD showed significantly elevated levels of both FBG (P<0.001) and RBG (P<0.001) when compared with that of the control group (Fig 1B and 1C, respectively), but at the end of treatment period, HES alone and/or in combination with ORL, exhibited almost progressive hypoglycemic effects on FBG and RBG when compared with that of ORL-treated group alone. Similarly, we also recorded the serum level of insulin (Fig 1D) before, during and at the end of treatment period; we found that HFD significantly (P<0.001) increased the serum level of insulin before the start of treatment period when compared with that of NC-group. Whereas, when the treatment started, we found that HES (P<0.001) improved the insulin sensitivity by decreasing the serum level of insulin. When HES was used in combination with ORL, it exhibited better effects (P<0.001) as compared to that of ORL alone (Fig 1D).

Before the end of treatment period, we also performed OGTT to determine and/or compare the effects of HES alone and in combination with ORL after an overnight starvation (Fig 1E). We obtained the blood to measure FBG and serum insulin before the administration of calculated amount of glucose according to the body weight of individual rat of experimental groups. Upon the administration of glucose, blood samples were collected at pre-defined time points to measure the blood glucose level. The highest level of blood glucose was achieved at 30 minutes in HFD-group as compared to the rest of all experimental groups (Fig 1E) and remained persistently high at all time points till at 120 min. The level of blood glucose in HES alone or in combination with ORL was comparatively lower (P<0.001) as compared to that of ORL-group (Fig 1E). Based on the fasting levels of blood glucose and serum insulin, we also evaluated the effect of treatment on insulin resistance with the help of HOMA-IR. We noticed that HES alone or in combination with ORL significantly improved HFD-induced insulin resistance when compared with that of ORL alone (Fig 1F).

### Assessment of treatment on lipidemia

**Fig 2:**
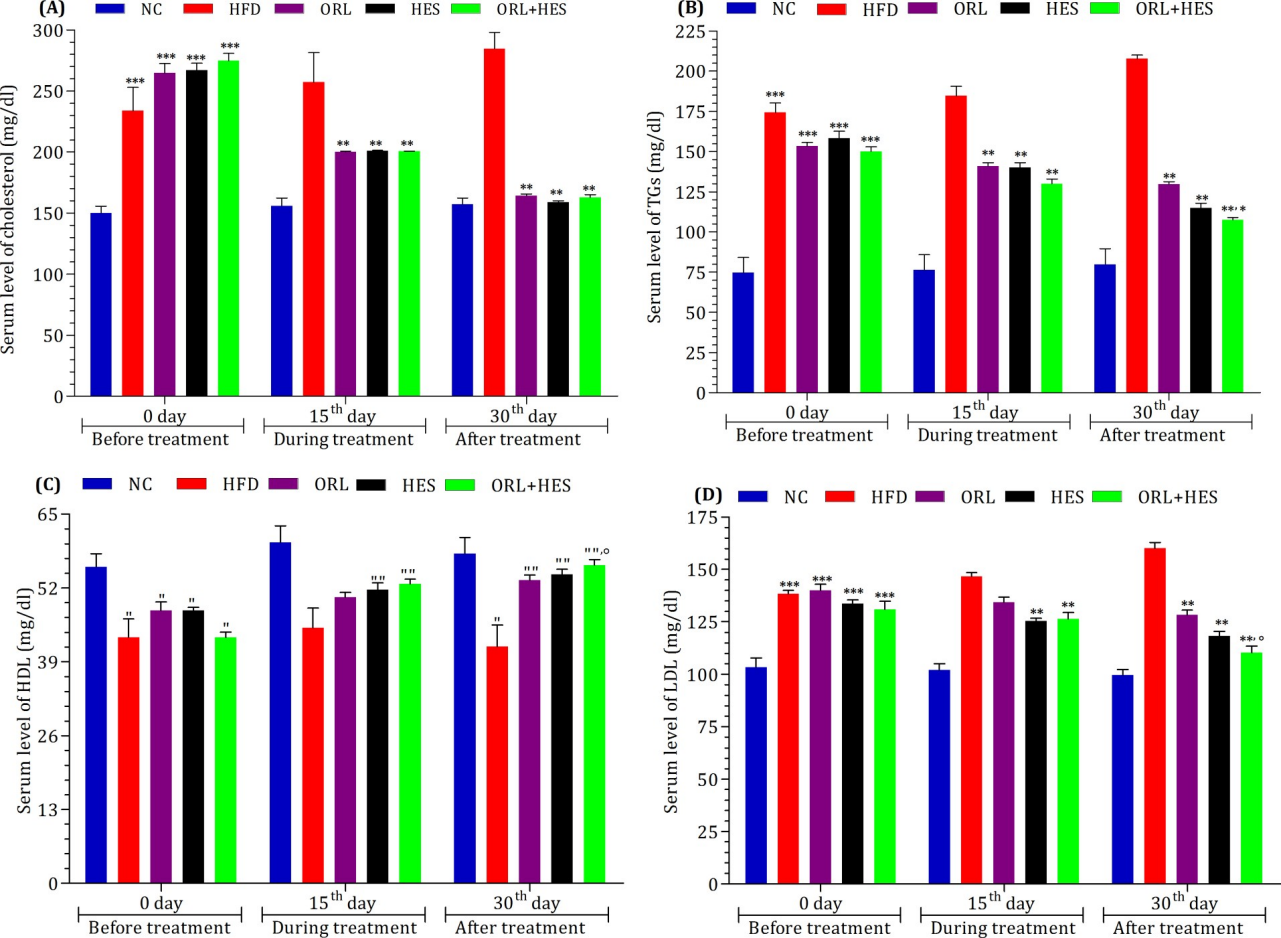
Effect of treatment on lipidemia. To determine the effect of HFD and treatment on lipid profile, serum level of (A) cholesterol, (B) TGs, (C) LDL and (D) HDL was measured at 1^st^, 15^th^ and 30^th^ day of the treatment period. The level of significant difference was estimated by Bonferroni post-test using two-way ANOVA. *** represent P<0.001 when compared with control group. ** represent P<0.001 when compared with HFD group. * represent P < 0.05 when compared HFD group. " represent P<0.05 when compared with control group. "" represent P<0.05 when compared with control group. ° represent P<0.05 when compared with ORL group.

Fig 2 briefly exhibits the effect of HES alone and/or in combination with ORL on lipid profile biomarkers notably cholesterol, TGs, HDL and LDL. When experimental rats were fed with HFD before the start of treatment, the serum levels of cholesterol, TGs and LDL were high (P<0.001), whereas, the serum level of HDL was low (P<0.05) when compared with that of NC-group (Fig 2). When treatment started, we found that HES alone or in combination with ORL improved the serum levels of these lipid profiles. Moreover, we observed the improvement in lipid profile after the treatment with ORL (*P*<0.05) and HES (*P*<0.01) alone when compared with that of HFD-group (Fig 2). Interestingly, the combination of both ORL and HES, however, showed an expressively (*P*<0.05) ameliorating effect on HFD-induced alteration in lipid profile as compared to that of ORL-treated group (Fig 2).

### Assessment of treatment on leptinemia and inflammatory responses

**Fig 3:**
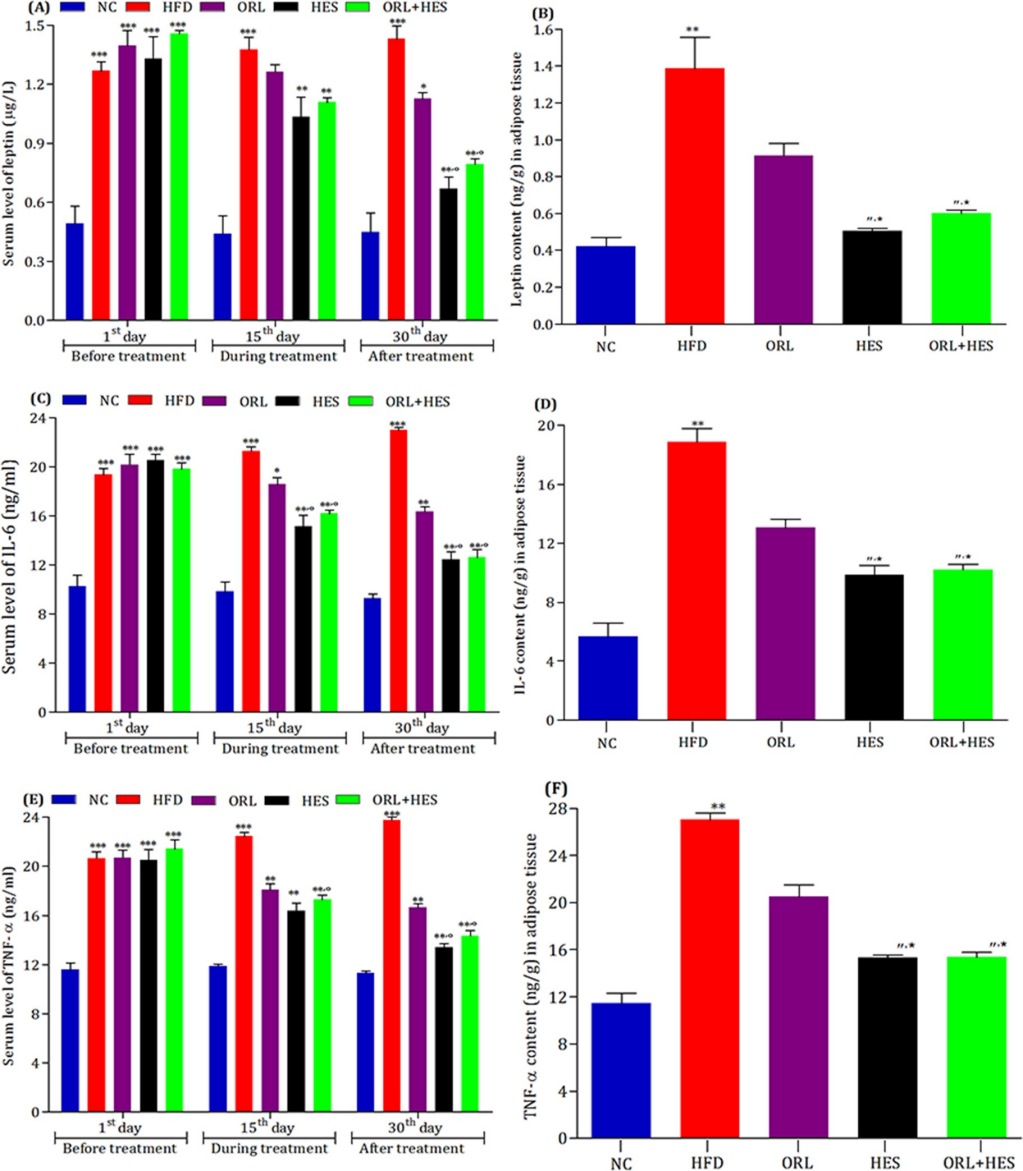
Effect of treatment on leptinemia and inflammatory responses. To estimate the effect of high-fat diet (HFD) and treatment on serum levels of (A) leptin (C) IL-6 and (E) TNF-α at 1^st^, 15^th^ and 30^th^ day, whereas, (B) leptin, (D) IL-6 and (F) TNF-α contents in tissue homogenate at the end of treatment period. For serum levels of leptin, IL-6 and TNF-α, the level of significant difference level was estimated by Bonferroni post-test using two-way ANOVA. *** represent P<0.001 when compared with control group. * represent P<0.05 when compared with HFD group. ** represent P<0.001 when compared with HFD group. ° represent P<0.01 when compared with ORL group. For leptin, IL-6 and TNF-α contents in tissue homogenate, the level of significant difference was estimated by Newman-Keuls multiple comparison test using one-way ANOVA. ** represent P<0.01 when compared with NC group. " represent P<0.01 when compared with HFD group. * represent P<0.05 when compared ORL group.

To determine the effect of HFD and treatment on serum levels and adipose tissue contents of inflammatory responses, we measured the levels of leptin, IL-6 and TNF-α in serum and their contents in adipose tissues (Fig 3). We found that HFD significantly increased (P<0.001) the serum levels of leptin (Fig 3A), IL-6 (Fig 3C) and TNF-α (Fig 3E) when compared with that of NC-group, but after the start of treatment, we found that HES comparatively exhibited the better effects by improving the serum levels of leptin, IL-6 and TNF-α when compared with that of HFD-group. HES in combination with ORL also improved the serum levels of these inflammatory mediators (leptin, IL-6 and TNF-α) in better way (P<0.01) than that of the ORL alone (Fig 3).

At the end of treatment, we also examined the effect of treatment on tissues content of leptin (Fig 3B), IL-6 (Fig 3D) and TNF-α (Fig 3F) in adipocytes of white adipose tissues. We found that HFD significantly (P<0.01) induced the secretion of leptin, IL-6 and TNF-α as compared to that of NC-group. Moreover, ORL and HES individually controlled the secretion of leptin (Fig 3B), IL-6 (Fig 3D) and TNF-α (Fig 3F), from the adipocytes of white adipose tissues as evident from the decreased levels of these inflammatory biomarkers in ORL- and HES-treated experimental animals when compared with that of HFD-induced obese animals. More surprisingly, the combination of ORL+HES controlled the secretion of leptin, IL-6 and TNF-α more efficiently (P<0.05) when compared with that of ORL-treated experimental animals, but a non-significant difference was found between HES- and ORL+HES-treated experimental animals (Fig 3B, 3D and 3F).

### Assessment of treatment on liver and kidney function biomarkers

**Fig 4:**
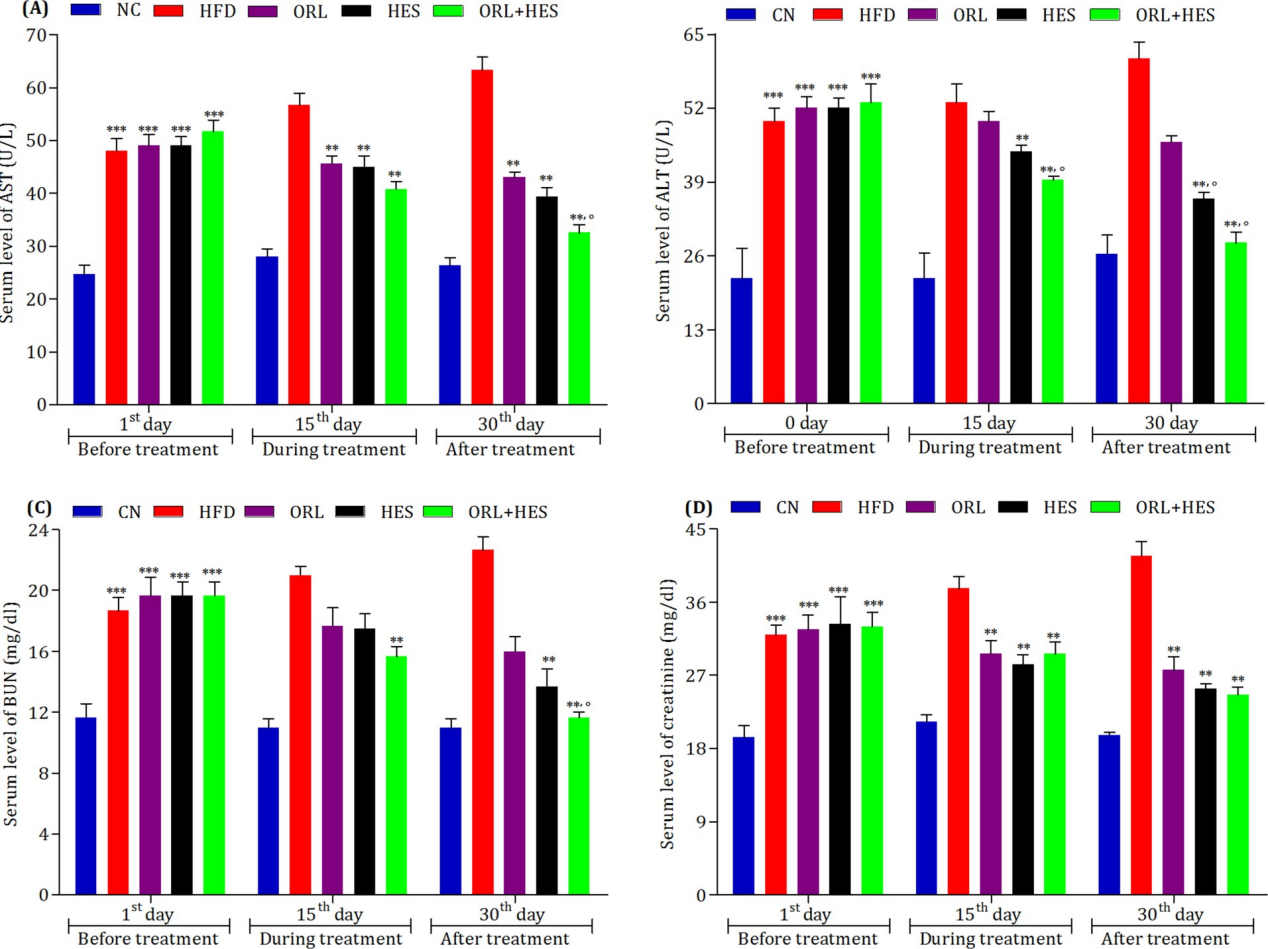
Effect of treatment on liver and kidney function biomarkers. To determine the effect of HFD and treatment on liver and kidney, serum levels of (A) AST, (B) ALT, (C) BUN and (D) creatinine were measured at 1^st^, 15^th^ and 30^th^ day of the treatment period. The level of significant difference was estimated by Bonferroni post-test using two-way ANOVA. *** represent P < 0.001 when compared with control group. ** represent P<0.001 when compared with HFD group. ° represent P<0.05 when compared with ORL group.

We assessed effect of HFD on the serum levels of AST and ALT in experimental animals and compared the effect of ORL- and HES-treatment alone and/or in combination with that of HFD-treated animals (Fig 4A and 4B). We found that HES (*P*<0.001), and ORL + HES (*P*<0.001) were responsible to decrease the elevation of these biomarkers when compared with that of HFD-treated animals. We also found a similar patter, when we compared the effect of ORL and HES (P<0.001) alone and/or in combination form (P<0.05) on the serum level of BUN (Fig 4C) and creatinine (Fig 4D) when compared with that of HFD-treated animals.

### Assessment of treatment on histology of body tissues

**Fig 5:**
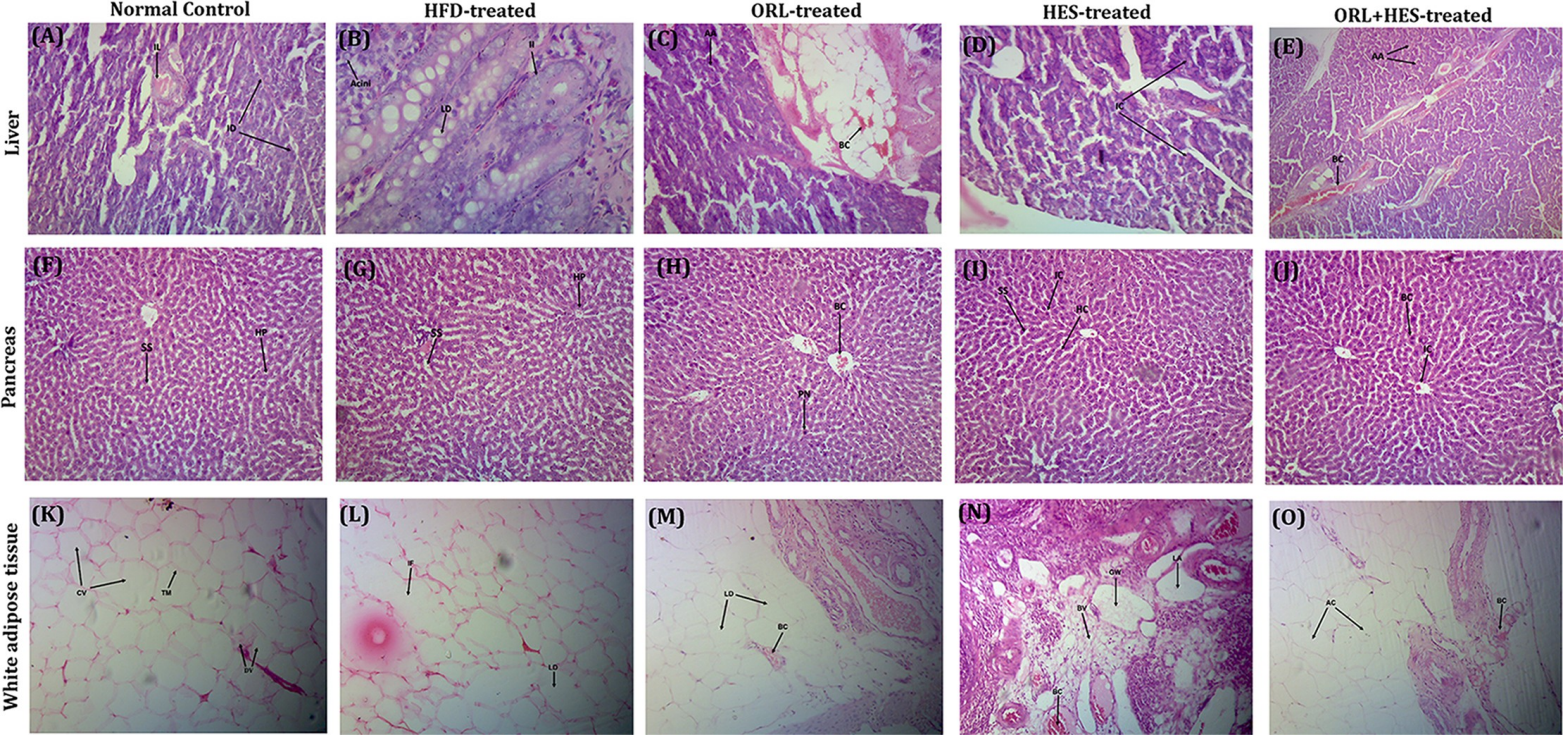
Histopathological examination of body tissues. **(A-E) liver**: (**A**) CN-group; Hepatic parenchyma (HP) is normal in appearance, hepatic cords arranged normally with prominent sinusoidal spaces (SS). Hepatocytes (HC) have prominent nuclei with normal chromatin. (**B**) HFD-group; Hepatic cords are not arranged in regular pattern. They are disrupted. HC indicating swelling having fatty changes with deposition of fat droplet inside cytoplasm. Infiltration of inflammatory cells (IC) at few places. (**C**) ORL-group; Bile duct hyperplasia (BDH) is present. Pyknotic nuclei (PN) is seen at few places indicating cell swelling and hepatoxic effects of orlistat were seen. Infiltration of inflammatory cells (IC) at few places indicating inflammatory changes. Blood congestion (BC) is present and prominent in most places. (**D**) HES-group; HC appearance is normal with prominent nuclei. Hepatic cords (HS) appearance is normal having normal SS. Presence of IC are seen at few places. (**E**) ORL+HES-group; Hepatic cords are normally arranged. Few HC are showing vascular degeneration along with cell swelling. BC is present at few places. Presence of IC are also seen at few places. **(F-J) Pancreas**: (**F**) CN-group; Intercalated duct (ID) is normal in appearance. Islet of Langerhans (IL) appears with normal pattern. (**G**) HFD-group; Lipid droplets (LD) are present indicating the fat deposition on pancreas. Slight injury was also seen in the acini. Islets inflammation (II) is also present. (**H**) ORL-group; Acinar atrophy (AA) was observed clearly. Blood congestion (BC) is present and clearly observed. Pancreatic degeneration was also observed. Inflammatory cells infiltration was observed in both islets of Langerhans and pancreatic acini. (**I**) HES-group; Islet of Langerhans appears normal. Inflammatory cells (IC) are seen at few places. (**J**) ORL+HES-group; Acinar cells (AC) are normal in appearance. Blood congestion (BC) is present at few places. Vascularization of islet of Langerhans (VIL) was also seen. Presence of inflammatory cells (IC) are also seen at few places. **(K-O) adipose**: (**K**) CN-group; Cytoplasm shows single and delimited vacuole (DV). The nucleus of tissue has central vacuole (CV) and there is presence of thin membrane (TM) between cells. (**L**) HFD-group; Increase in the size of the lipid droplets (LD) is seen. Adipocytes shows cytoplasm ring surrounding the lipid droplet having nucleus within the cells. Inflammation (IF) of the adipose tissue is also seen. (**M**) ORL-group; Reduction in the size of adipose tissue (AT) is seen. Congestion (BC) is also seen surrounding the tissue. (**N**) HES-group; Lobules of adipose tissue (LA) is seen. Blood vessels (BV) are also seen at few places. Gliotic white matter (GW) is also present. Congestion (BC) is seen at few places. (**O**) ORL+HES-group; Prominent changes are seen in the size reduction of adipocytes (AC). Congestion (BC) is seen at few places. Thin membrane (TM) is also seen between cells.

Fig (5A–5E) depicts the differences in the pattern of histological appearance of liver in all experimental groups. In control group, the liver section showed normal appearance of hepatic parenchyma and hepatic cords. Cells have prominent nuclei with normal chromatin. However, in HFD-group, there is a disrupted pattern of hepatic cords. Cells are showing swelling with deposition of fat droplet inside cytoplasm. Infiltration of inflammatory cells is also present at few places. However, the intervention with orlistat (Fig 5C), there is still presence of bile duct hyperplasia along with cell swelling and hepatotoxic effects at few sites. Nevertheless, there was an overall improvement in terms of effect of orlistat on hepatic parenchyma and hepatic cords when compared to HFD-group. When exposed to HES, there was a significant improvement in the histological appearance of liver with less swelling and infiltration in hepatocytes with prominent nuclei (Fig 5D). Hepatic cords were also seen with normal sinusoidal spaces. Likewise, ORL + HES group showed improved histological appearance (Fig 5E). Likewise, a similar pattern of histological appearance of pancreas (Fig 5F–5J) and white adipose tissues (Fig 5K–5O) in all experimental groups were seen before and after treatment.

### In silico studies

#### Molecular modeling and protein structures preparation

Crystal structures of LBD and leptin protein (PDB ID: 3V6O [31] and 1AX8 [42], respectively) have been solved and can be retrieved from RCSB Protein Data Bank (http://www.rcsb.org). Occasionally, the PDB files may have some missing residues, for which the coordinates were remained to be undetermined. For these two structures, 3V6O misses loop residues 452–459, 517–519, 559–566 and 591–593, whereas, 1AX8 misses loop residues 25–38 (S1 Fig). Although, these residues are distant from the active/binding site, they may influence overall protein flexibility. Hence, the tertiary structures of LBD and leptin proteins were predicted using the web server I-TASSER and the top ranked structures with the maximum confidence score (C-score = LBD (-1.28) and leptin (-0.25)) were selected for subsequent in silico studies. Structure quality of generated proteins was assessed using Ramachandran plot (S2 Fig). Moreover, the model quality was further verified by superimposing the I-TASSER generated model and their respective co-crystal structures (S1 Fig).

#### Protein-protein docking

At first, the best predicted models were submitted to MD simulations for 10 ns to obtain more refined and stabilized protein structure. Protein-protein docking studies were carried out HADDOCK online webserver. Ten models were obtained, only the model with highest binding affinity score (-152.8 +/-5.1) along with buried surface area (1273.0 +/- 66.1) was selected. HADDOCK computed binding energies and RMSD values are summarized in S1 Table.

#### Protein-ligand docking

In the present work, MD simulations were performed to study the key molecular interactions essentially responsible for difference in binding affinities of inhibitors (ORL and HES) bonded with LBD-LPT complex. All protein-ligand docking simulations were executed using the Surflex-Dock module of SYBYL-X 1.3 [33]. The docking scores (C-score) of ORL and HES for LBD-LPT complex are 8.01 and 10.73, respectively. However, as compared to HES (HES-LBD-LPT), ORL displayed relatively weaker binding affinities in ORL-LBD-LPT complex system. To provide more extensive insight into the protein-ligand interactions, the binding energies (consensus scores) and key residues involved in hydrogen bond interactions are tabularized in S2 Table.

**Fig 6:**
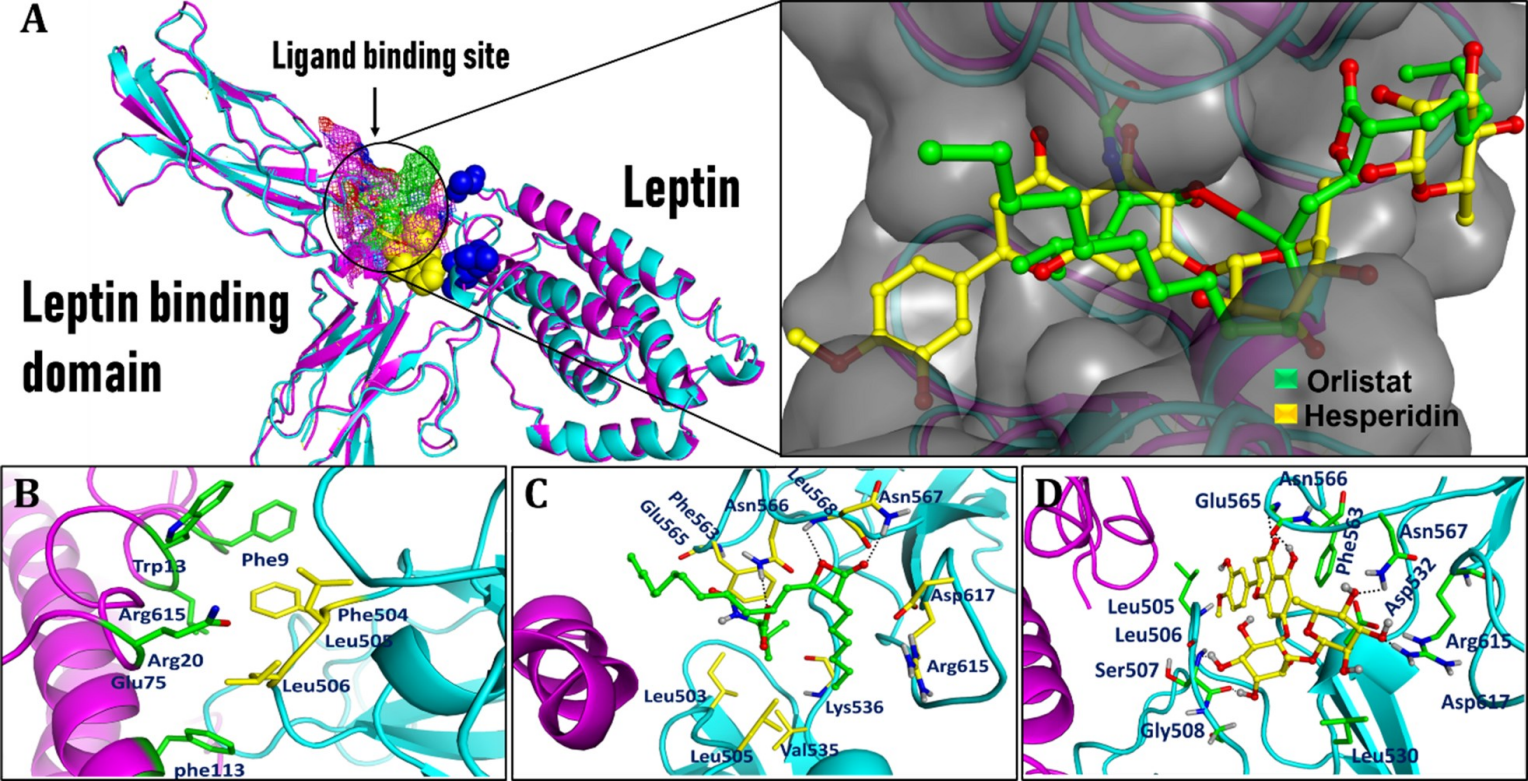
Obtained protein-protein and ligand-protein docking simulated conformations. (A) Superimposing of compounds; ORL (Green) and HES (Yellow) docked to LBD-LPT complex. (B) Protein-protein docked model among LBD (cyan) and LPT (magenta) (C) Binding mode of compound ORL in LBD main binding site. (D) Binding mode of compound HES in LBD main binding site.

The top-ranked docking simulated conformations of ORL and HES in LBD-LPT complex systems were saved and graphically examined to reveal the ligand–protein mode of interactions. Docking results show that both ligands occupy the same cavity in LBD (Fig 6) situated at the interphase of LPT binding site and establish molecular interaction with key residue in a similar fashion. As depicted in Fig 6C and 6D, Phe563, Asn566, Asn567, Leu568, Arg615 and Asp617 are the most important residues taking-part in ligand-protein interactions in both ORL- and HES-LBD-LPT complex systems. The involvement of these residues has already been reported in making key interactions with ligands [31, 32], which supports the accuracy of docking results of our study. The optimal energy conformations of ORL and HES in LBD-LPT complex system are depicted in Fig 6, and the concrete H-bonds and its corresponding bond lengths are listed in S3 Table.

#### Molecular dynamics simulation

**Fig 7:**
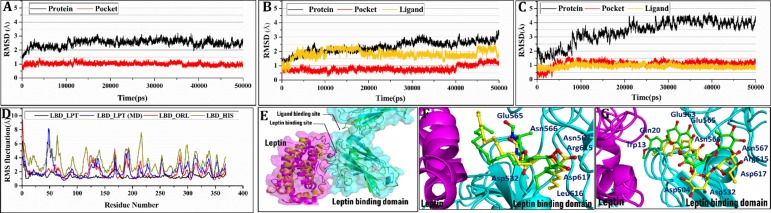
RMSDs of the receptor (Cα atoms) binding pocket (backbone atoms), and the ligand (heavy atoms) for (A) LBD-LPT, (B) ORL-LBD-LPT, (C) HES-LBD-LPT, (D) Comparison of residual flexibility profile between crystallographic LBD-LPT complex (black) and thee LBD-LPT complexes; LBD-LPT(blue), LBD-ORL (red), and LBD-HES (green) during a 50-ns MD simulation, as calculated by RMS fluctuation (RMSF), presented higher fluctuations in the LBD-HES than in LBD-LPT complex. (E) Superimposed structures of LBD-LPT (cyan-magenta) before and after (green-yellow) 50-ns MD simulations (F) ORL-LBD-LPT (green) before (yellow) after (G) HES-LBD-LPT (green) before (yellow) after 50-ns MD simulations.

Despite the fact that docking simulations can provide quite reasonable binding mode, the solvent, density, pressure and temperature effects remain to be unconsidered. Therefore, the docking generated complexes (LBD-LP, ORL- and HES-LBD-LPT) were post-processed with more reliable MD simulation for 50 nanoseconds in explicit aqueous solution. To examine the dynamic stability of complexes and rationalize the results of MD simulation analysis, the backbone stability of whole protein and active site was assessed in terms of root-mean-square deviations (RMSD) over entire 50 nanosecond of MD trajectories. Fig 7A–7C illustrates RMSD curves for proteins, active site, and ligand for the entire MD simulated snapshots in comparison to the initial conformation of each corresponding systems. In short, all complex systems remained stable and no deviation beyond 2.5 Å were observed for pocket or ligand throughout the simulation. Fig 7D shows that each RMSD of LBD residues from MD trajectories are in good agreement with the results derived from experimental crystallographic data. The protein structures of all three complexes demonstrate similar fashion of RMSF plots to their starting structure. The higher RMSD values of active site residues in ligand bonded complexes reflects the impact of ligand binding. Overall, the RMSD analyses suggest that docking simulated conformations are quite reasonable and stable enough to be used for binding free energies calculation.

#### Binding free energy analysis

**Fig 8:**
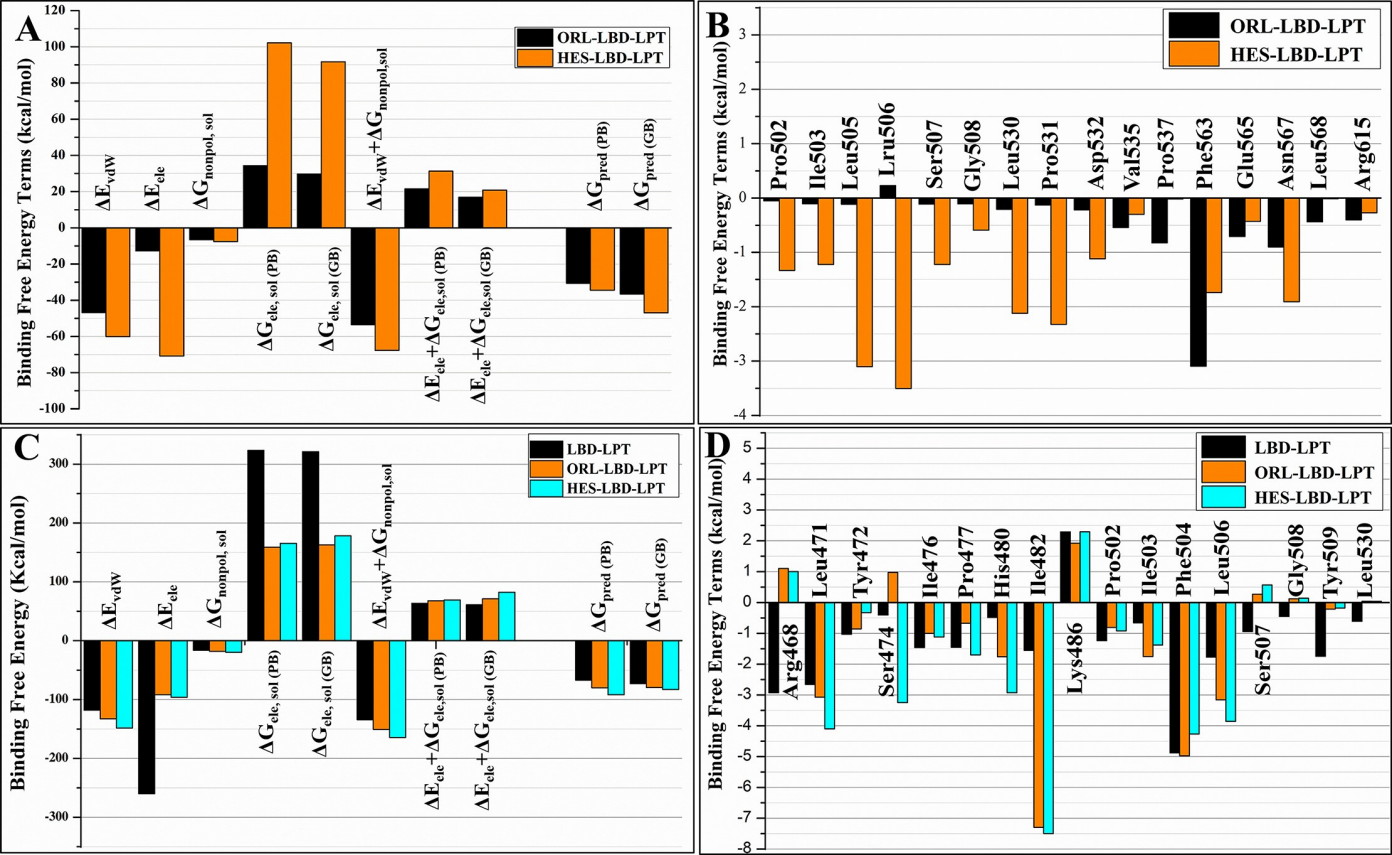
Comparison between binding free energy terms of protein-ligand and protein-protein complexes. (A) ORL- and HES-LBD-LPT (B) protein-protein (LBD-LPT) ligand bonded (ORL-and HES-LBD-LPT). Per-residue energy decomposition analysis for protein-protein and protein-ligand systems (C) ORL- and HES-LBD-LPT (D) protein-protein (LBD-LPT) ligand bonded (ORL-and HES-LBD-LPT).

The binding free-energies of ORL and HES bonded to LBD-LPT complex were calculated using the MMGB/PBSA approach. The results are plotted and summarized in Fig 8A and S4 Table, respectively. The results demonstrate a noticeable difference of the computed binding affinities for compound ORL (Δ*G*_pred (GB)_ = -36.58 kcal/mol) and HES (Δ*G*_pred (GB)_ = -46.97 kcal·mol^−1^) bonded to LBD-LPT system. This significant difference in binding free energies sufficiently reflects the fact that HES exhibit greater binding affinity towards LBD-LPT than ORL. Furthermore, per-residue energy decomposition analysis reveals that the residues binding to HES in LBD-LPT complex share more negative binding free energy values than that of corresponding residues in ORL-LBD-LPT complex (Figs 8B and S5 Table). These findings are in good agreement with molecular docking results and suggest that the compound HES binds more tightly to the LBD than that of ORL. Additionally, the binding free energies for protein-protein complex formation was also calculated using MMGB/PBSA approach. The results indicate an appreciable increase in binding free energies in LBD-LPT complex upon ligand binding (Fig 8C and S6 Table).

## Discussion

Insulin resistance has long been known to significantly increase the leptin production in adipocytes [43]. HDF is responsible to increase the level of leptin in serum that may further reduce the transportation of leptin through the blood-brain barrier [44]. During leptin resistance, there is an increased secretion of leptin from white adipose tissue, but this leptin does not bind with its receptor (LEP-R) present on hypothalamus in brain, causing resistance to the leptin and increasing the body weight which leads to the obesity [1]. Various factors are considered responsible for this unavoidable condition of hyperleptinemia [45]; likewise, different conventional drugs are being used for its management including inhibitor of pancreatic lipase, like orlistat. This drug may act to decrease the body weight by reducing the absorption of fat [46]. However, few adverse effects like, liver toxicity and steatorrhea has been reported with this drug.

We have therefore investigated the therapeutic potentials of hesperidin, which has been known for wide range of therapeutic responses. These responses include anti-oxidant, anti-inflammatory, decrease fat accumulation and better metabolism of the lipids [47]. However, the role of hesperidin on hyperleptinemia and HFD-induced leptin resistance is still unclear and needs to be elucidated. In this study, we have attempted in to elucidate this unrevealed effect of hesperidin alone and in combination with well-known anti-obesity drug, orlistat using HFD-animal models. Our results have demonstrated that hesperidin alone and in combination with orlistat have significant effects on reducing body weight (Fig 1A) and in improving glycemic profile (Fig 1B–1D) as compared to that of the orlistat treated experimental rats. Alongside with obesity, insulin resistance and glucose intolerance are considered major factors in charge for obesity-related complications [48–50]. These two aspects are majorly accountable for augmented production of glucose in liver, more glycemia in blood, where insulin secretion is impaired from β-cells of pancreatic islets and glucose intake in muscle is also decreased [51]. Hence, we performed OGTT and HOMA-IR to predict insulin resistance and glucose tolerance respectively. In Fig 1E and 1F, the results clearly depicted the potential of hesperidin solely for improving the glucose tolerance (Fig 1E) and reducing insulin resistance (Fig 1F).

Abnormal fat metabolism is one of the main causative factors for obesity [52, 53]. In our study, we found that HFD significantly increased (P<0.001) the serum levels of cholesterol, TGs and LDL whereas, the serum level of HDL was decreased in HFD-treated animals (Fig 2). The treatment of hesperidin showed its potential for lowering the serum levels of cholesterol, TGs and LDL, whereas the serum level of HDL was improved. These results were found to be corresponding in terms of anti-lipidemic effects of many plants oriented bioactive flavonoids [40, 54].

Recently, it has been obvious that inflammation is one of the major hallmarks for the pathogenesis of DM and development of insulin resistance [48, 49, 51, 55–59]. Among the various inflammatory mediators, leptin, IL-6 and TNF-α are the major pro-inflammatory mediators that paly their decisive role to induce the inflammatory responses during the pathogenesis of DM and development of insulin resistance [1, 49, 57, 60]. In this study, we also focused on the effect of HFD, ORL and HES on the serum level and tissue contents of leptin, IL-6 and TNF-α (Fig 3). HFD increased the leptin resistance as evident from the elevated levels of leptin in serum (P<0.001) and white adipose tissues (P<0.01) when compared with that of NC-group animals (Fig 3A & 3B). Whereas, HES decreased the levels of leptin in serum (P<0.01) and white adipose tissues (P<0.05) more efficiently even when compared with that of ORL-treated experimental animals. HES also decreased the levels of IL-6 (Fig 3C & 3D) and TNF-α (Fig 3E & 3F) in serum and white adipose tissues when compared with that of ORL-treated experimental animals. It has been reported that better leptin binding with its receptor may result leptin as an anti-obesity hormone [61]. Hence, this combination therapy of hesperidin with orlistat show to have therapeutic potential against leptin-resistance and its associated changes. This combination may be helpful in future for patients having metabolic disorders especially obesity involving hyperinsulinemia, hyperleptinemia and corresponding resistances.

AST and ALT are very critical biomarkers reflecting the normal functioning of the liver. Elevated levels of these enzymes in serum, shows abnormal functioning of the liver. In HFD-induced obesity, the increased level of AST and ALT may indicate the uncontrolled and irregular metabolic function of the liver [40, 54]. In present findings, hesperidin alone and in combination with orlistat has shown to regulate the serum levels of AST (P<0.001) and ALT (P<0.001) when compared with that of HFD group (Fig 4A and 4B). Correspondingly, hesperidin alone and in orlistat combination has also shown to improve the HFD-influenced kidney function biomarkers (Fig 4C and 4D) The effects of hesperidin alone and/or in combination on functional biomarkers of the kidney and/or liver also relate with some of the previously reported studies [22, 62, 63].

The results of histological examination of pancreas, liver and white adipose tissue (Fig 5) were also found to be in accordance with the biochemical results of this study. HFD has shown prominent degeneration of hepatocytes as revealed on histological study. Pyknotic nuclei, an irreversible condensation of chromatin in cell nucleus having apoptosis or necrosis were also seen in case of HFD having fragmentation of the nucleus. There was extracellular matrix, which degraded slowly and was overproduced; this might be a factor triggering chronic injury [14, 64, 65]. However, these damaging effects on liver histology were in accordance to the biochemical results of our study stated above showing improvement upon treatment hesperidin alone and in combination with orlistat. Similar patterns were observed for pancreatic and adipose tissue histological appearances among non-treated and treated groups. This may be because of the fact that HFD mainly results in inflammation [66]. Interestingly, it has been confirmed in few works using brain endothelial cell lines and co-culture model that hesperidin and it’s in vivo circulating metabolites can cross the blood-brain barrier [67]. We used combination agents; orlistat and hesperidin to focus on simultaneous targets of more than one biological mechanisms (other than that of orlistat) including improved status of glycemia and anti-inflammatory action beside resolving leptin resistance and regulating lipid profile. Moreover, such combinations might ultimately be more effective in producing sustained weight loss and improvements in comorbidities.

**Fig 9:**
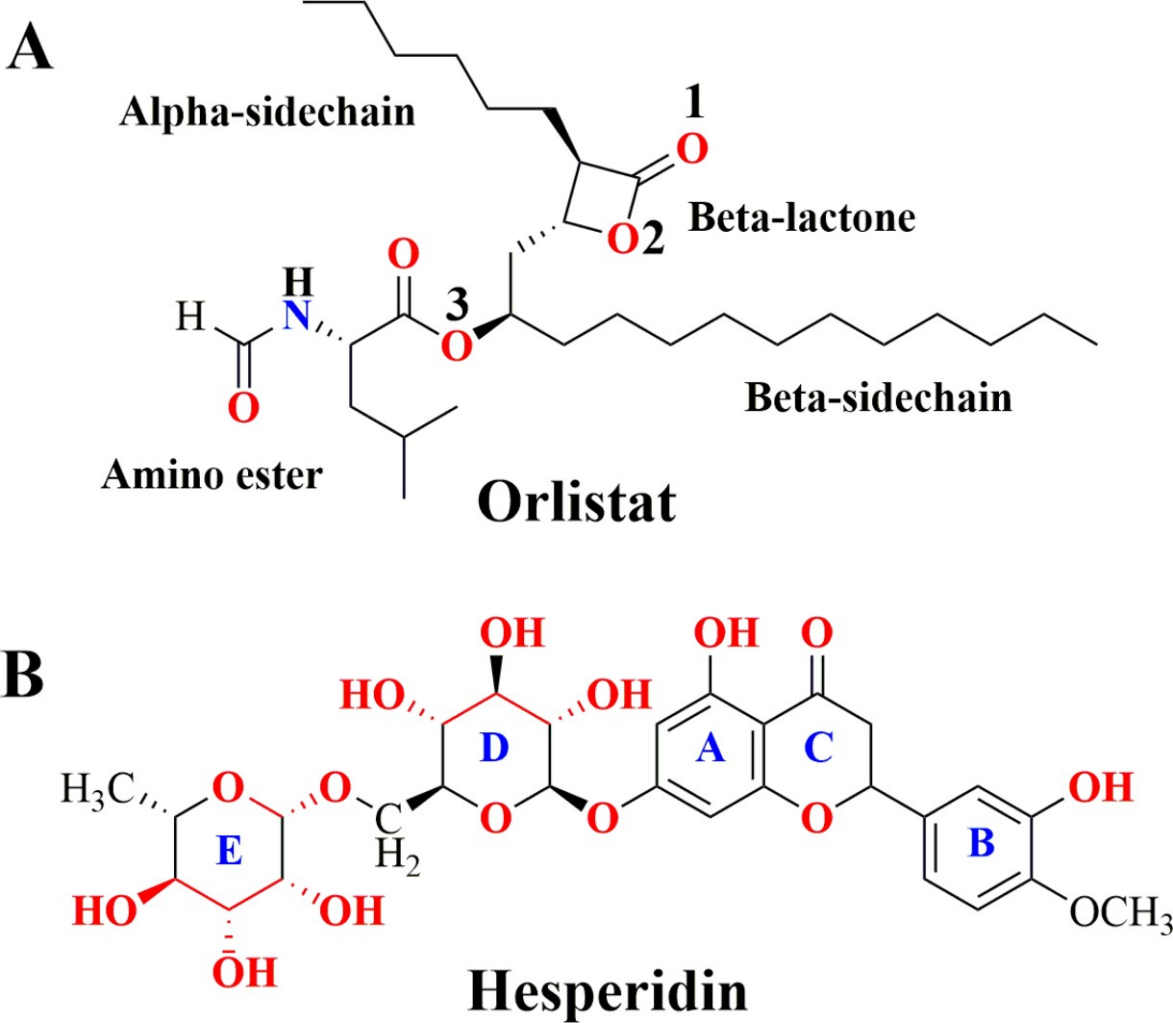
Molecular structures of Orlistat (A) and hesperidin (B).

As far as the *in-silico* study in terms of molecular modelling using crystal structures of LBD and leptin protein (S1 Fig) is concerned, structure quality of generated proteins was assessed using Ramachandran plot. This plot (S2 Fig) revealed that 98.2% and 97.3% of residues were in the allowed region for LBD and leptin protein, respectively. Moreover, superimposing showed that the modeled structures of LBD and leptin protein were almost overlapped to their crystallographic conformations with RMSD value less than 0.74 Å and 0.61 Å, respectively. Moreover, both proteins shared well-folded and native-like geometrical conformation. Similarly, for protein-protein docking among complete models of LBD and LPT, their overall binding affinity were calculated and the residues critically involved in LBD-LPT binding were identified. According to the docking results, residues Glu484, Leu471, Arg468, Phe504, Leu505 and Leu506 of LBD and Arg15, Arg20, Glu75, His109, Phe113, His118 and Trp121 are directly involved in LBD-LPT complex formation (Fig 6A and 6B). Interestingly, the residues of LBD (Phe504, Leu505, and Leu506) and LPT (Arg20, and Glu75) are critical because the involvement of these residues has already been reported in making key interactions in LBD-LPT complex [31, 32], which further supports the accuracy of docking results of present study. However, when Protein-ligand docking was carried out in the present work, the docking scores (C-score) of ORL and HES for LBD-LPT complex were found to be 8.01 and 10.73, respectively, which indicates that both ligands exhibits strong binding affinity towards LBD-LPT complex (S2 Table). However, ORL may just form three H-bonds with residues i.e. Asn566, Asn567 and Leu568 in ORL-LBD-LPT system (Fig 6C). Unlike HES, the perceptible σ-π and/or π–π stacking does not exist between orlistat and hydrophobic or phenyl side chain of corresponding residues (S3 Fig), as ORL lakes an aromatic ring in its structure (Fig 9). Hence, one can conclude from molecular docking results that the magnitude of HES binding affinity towards LBD is slightly higher than the ORL. Definitely, this increased binding affinity is the consequence of additional H-bonding, σ-π and π–π interaction of HES with surrounding residues. Moreover, a comparative analysis of interaction modes of ORL and HES in their respective complex systems has revealed that HES occupies the major area of binding site to make at least six H‐bond interactions with nearby residues such as Ser507, Gly508, Glu565, Asn566, Asn567 and Arg615. In addition to H-bond interactions, the conjugated effect has also been found in the HES-LBD-LPT system. Definitely, the plane of the benzene moiety (A-ring) in HES lies parallel to the plane of the benzene ring of nearby residue Phe563 (3.21 Å), which indicated the presence of π–π stacking between LBD and hesperidin. Moreover, the methoxy-phenol moiety (B-ring) extends deep into a shallow hydrophobic cavity to establish an additional σ-π stacking with side chain of Leu505.

For MD simulation, the pattern of RMSD curves indicate that after 10 to 15 ns of MD simulation each system rapidly achieved equilibrium, which indicate stable protein-protein and ligand-protein conformation for docked structures. Fig 6 also elaborates the RMSD of the ligand (in yellow) in the binding cavity, where average RMSD value of ORL arises to ∼1.8 Å during 5 to 15 ns, then level off to ∼1.2 Å in the following 30 ns, whereas the average RMSD value of HES remains constant at ∼0.7 Å during the entire 50-ns of simulation. These results indicate that HES acquire relatively more stable conformation in HES-LBD-LPT system than ORL in its corresponding complex. However, it was not surprising as the chemical structure of HES is relatively less flexible than ORL where a two flexible hydrocarbon chains are substituted to a lactone moiety. Therefore, ORL side chains may switch between different kinds of conformations in the active site, which may lead to disruption of various *vdW* and hydrophobic contacts with surrounding residues. These conformational drifts were further verified by superimposing the MD-simulated averaged coordinates of ligand bonded and unbonded LBD-LPT complexes over their respective initial confirmations (Fig 7E–7G). These findings also provide clue regarding higher binding affinity of HES over ORL in LBD-LPT complex system. All these data validate the reliability of the MD results. In Fig 7D, the RMSF plots for ORL and HES bonded systems display quite similar but relatively higher fluctuations, which indicate that both inhibitors occupy the same cavity and produces significant impact on overall structural conformation of LBD-LPT system. The noticeable difference between RMSF values of ligand bonded and unbonded complexes indicate that both compounds may induce tremendous conformational drift in LBD structure to negatively influence LBD-LPT complex formation. Furthermore, among all three complexes the highest RMSF fluctuations have been observed in HES bonded system, implying that HES-LBD-LPT had a comparatively higher structural mobility than the other two complexes.

Last but not the least, a comparison between the computed binding affinities of ORL and HES towards LBD-LPT complex system was performed by utilizing MM/PB(GB)SA approach. The ranking of the computed binding free energy values ΔG_pred (GB)_ reveals that the compound HES (-46.97 kcal·mol^−1^) is more strongly bonded to LBD-LPT complex than ORL (-36.58 kcal·mol^−1^). Similarly, the free energies (ΔG_pred (PB)_) computed by MM/PBSA approach also demonstrate the similar ranking of binding affinities; which indicate that the compound HES interact more efficiently to LBD-LPT (-34.51 kcal·mol^−1^) than ORL (-30.66 kcal·mol^−1^). Encouragingly, the predicted binding free energies are in strong agreement with molecular docking and experimental findings. The considerable difference in binding free energies reflect the fact that the compound HES is the more potent LBD antagonist than ORL. In order to get more detailed insight into the deriving forces responsible for difference in binding affinities of ligands bonded to the same binding site; the cumulative MM/PB(GB)SA binding free energies were decomposed into independent energy components. A comparison of individual binding free energy components for ORL and HES bonded systems (Fig 8A and S4 Table) indicate that the van der Waals (*vdW*) and nonpolar solvation energies (ΔE_*vdW*_+ΔG_nonpol,sol_) favorably contribute in ligand-receptor complex formation. As shown in Fig 8A, the *vdW* and the nonpolar solvation energy contributions originating either from π–π/σ–π stacking and/or the burial of the hydrophobic moieties (A and B ring) are the favorable binding‐free energies for HES (−60.11 kcal·mol^−1^) over ORL (−46.86 kcal·mol^−1^). Although, the favorable electrostatic energies (gas phase) are counteracted by the unfavorable polar solvation (ΔG_ele+pol_) energies; the electrostatic interactions have been identified to be the major contribution (-70.87 kcal·mol^−1^) in HES bonded system. Whereas, in case of ORL bonded system, a significantly decreased electrostatic contribution (-12.82 kcal·mol^−1^) has been observed. These findings suggest that; although the difference in ΔE_*vdW*_+ΔG_nonpol,sol_ contribution may remarkably influence the binding affinity of the HES towards LBD-LPT system, the decrease in electrostatic contribution is key component to be responsible for the tremendous decrease in binding affinity of ORL. Furthermore, according to the molecular docking results, ORL was able to establish only three H-bond interactions upon binding with LBD-LPT system. Whereas, HES establishes at least six H-bond interactions with surrounding residues in HES-LBD-LPT complex (Fig 6C and 6D). These results further reinforce the rationality of MD simulation results. Thus, it might be speculated that the electrostatic interactions, polar solvation free energies and H-bond interactions are the key driving forces responsible for dominant binding affinities of HES in its respective complex.

In order to identify key residues potentially involved in the ligand-receptor complex formation, the absolute binding free energy (MM/GBSA) was decomposed into per-residue energy contribution (Fig 8B). In addition, a comparison of per-residue (ΔG_ligand-residues_) interaction energy contribution of the active site residues in terms of *vdW* (Δ*G*_v_) and electrostatic (Δ*G*_e_) components for both of ligand bonded systems (ORL- and HES-LBD-LPT) are summarized in S5 Table. As shown in Fig 8B, the major favorable contributions to the ligand-receptor complex formation predominantly arise from the residues Pro502, Ile503, Leu505, Leu506, Ser507, Leu530, Pro531, Asp532 and Asn567 in HES bonded system, and the corresponding residues Val155, Pro537, Phe563, Glu565, Leu568, and Arg615 in ORL bonded system. Furthermore, the energy decomposition analysis has identified six key residues, Leu506, Ser507, Asp532, Glu565, Asn567, and Arg615 to be critically involved in making favorable electrostatic contacts with HES bonded to LBD-LPT system. Results obtained from molecular docking studies reveal that all of these residues lie in close proximity to HES; where they may establish several H-bond interactions in donor-acceptor motif. Moreover, Leu505, Leu530 and Pro531 represent favorable hydrophobic and *vdW* contribution in ligand receptor binding (S5 Table). As depicted in Fig 4B, almost all residues contributed for HES binding to LBD-LPT; also, favorably contribute for ORL-LBD-LPT complex formation. Unlike HES-LBD-LPT complex, the residues Pro502, Ile503, Leu505, and Leu506 are not found to be energetically valuable in ORL-receptor complex formation. The binding ORL to its receptor is potentially mediated by five residues namely Val535, Pro537, Phe563, Glu565 and Leu568 (Fig 8B). These outcomes clearly indicate that the compound HES interact with greater number of residues in the active site of LBD to establish various electrostatic and *vdW* interactions which were found to be missing in case of ORL bonded system.

Moreover, the study was further extended by employing MM/PB(GB)SA approach to calculate the binding free energy for the association of two proteins (LBD and LPT). The idea behind this extension of work was to investigate; whether, the ligand binding to LBD-LPT complex strengthens or weakens the protein-protein interaction. Keeping in view the RMSD and RMSF plots, it was assumed that the ligand binding to receptor with higher affinity may potentially influence protein-protein interactions. To inspect the variations in protein-protein (LBD-LPT) interaction energies upon ligand (ORL and HES) binding; a comparison of ΔG_protein-protein_ between ORL- or HES-bonded and non-bonded systems was performed (Fig 8C, S6 Table). The results demonstrate a significant increase in protein-protein binding affinities for ligand bonded systems (ΔG_pred (GB)_ = -79.52 kcal/mol, ORL-, ΔG_pred (GB)_ = -82.75 kcal/mol, HES-receptor) as compared to non-bonded systems (ΔG_pred (GB)_ = -72.81kcal/mol). Fig 8C depicts that the values of Δ*E*_ele_ for ligand unbonded system (-259.97 kcal/mol) are dramatically higher than that of ligand-bonded complexes (-91.60 and -96.24 kcal/mol). Inversely, the polar solvation energies (Δ*E*_ele,sol_) share positive values for all three systems to counteract with the favorable contribution of electrostatic energies (Δ*E*_ele_) in gas phase. Thus, the sum of the electrostatic and polar (Δ*E*_ele_+Δ*G*_ele, sol_) contributions in vacuum and solvent seems to be unfavorable for protein-protein complex formation. Whereas, the sum of *vdW* interactions and the nonpolar solvation energy (Δ*E*_vdW_ + ΔG_nonpol,sol_) are negative in all three complexes to be favorable for protein-protein binding. Such a finding is not surprising as there are a several hydrophobic residues, including Leu, Ser, Ile, Pro, Phe, Gly constituting the the leptin-binding site in leptin receptor. Furthermore, the results (Fig 8C) indicate that the values of Δ*E*_vdW_ for HES-bonded system are highest among all studied systems. Therefore, it could be concluded that the vdW and nonpolar interactions are responsible for improving protein-protein binding affinities in LBD-LPT systems. Therefore, it could be speculated that the vdW and nonpolar interactions are the key driving forces in LPT binding to its binding site in LBD and the conformational changes induced by ligand-binding may enhance these interactions to strengthen the protein-protein interactions in LBD-LPT complex. The energy contributions of each residue in protein-protein (LBD-LPT) binding process are illustrated in Fig 8D. The results indicate that the same residues which contributed for ligand unbonded LBD-LPT complex formation, also contribute in ligand bonded system. However, an appreciable increase in overall energy contribution of residues Leu471, Ser474, Pro477, His480, and Leu506 in ligand bonded system is observed. Meanwhile, Gly508, Tyr509, and Leu530 make relatively weak contacts in HES-bonded systems than non-ligand-bonded system. Taking in view the results of RMSF and graphical analysis; it can be spatulated that the variation in ΔG_protein-residue_ is the consequence of spatial variation of key residues and changes in binding site geometry induced by ligand binding. These observations are in agreement with our experimental results and RMSF plots. The strongest protein-protein interaction has been observed in HES-LBD-LPT complex system which coincide with our speculations; as HES holds a relatively bigger structure (Fig 9) predominated by cyclic moieties. Thus, upon binding, HES may induce spatial and geometrical variations in LBD structure which may favorably enhance LBD-LPT complex formation. These significant findings would help to recognize the potential structural and pharmacophoric features governing the process of complex formation and may provide considerable guideline for future drug design. Moreover, increased leptin level and adiposity may produce an indication to decrease food intake and intensify energy expenditure [68] this may accompany with fat lipolysis for reduction in serum lipid levels after treatment with hesperidin.

## Conclusion

Consequently, improvement of leptin and insulin resistance by hesperidin treatment alone and/or in combination with orlistat might be in part by improvement in serum and tissue levels of leptin and insulin respectively as found in results. Hence, we conclude that hesperidin alone and/or in combination with orlistat shows therapeutic activity against HFD-induced alteration in obese experimental animal model. In silico studies have uncovered the fact underlying the enhanced leptin-leptin receptor dimerization upon ligand binding. The energy difference in the molecular docking integrated with MD simulation analysis for LBD-LPT and LBD-LPT complex with ligand (HES or ORL) shows that in HES bonded system, LPT is more strongly associated with LBD rather than isolated form. On the basis of findings, one may propose that the dramatic increase in energy contribution of residues Leu471, Ser474, Pro477, His480, and Leu506 might be originated from spatial and geometrical variations in LBD structure due to incorporation and binding of ligand.

## Supporting information

S1 Fig 3D structures of proteins. Built by I-TASSER (magenta) superposed over X-ray crystal structure of corresponding proteins (cyan). The loops constructed with molecular modeling are highlighted in yellow color.
(TIF)

S2 Fig Ramachandran plot analysis. It was performed with RAMPAGE online webserver **(A)** leptin binding domain of leptin receptor and **(E)** leptin.
(TIF)

S3 Fig 2D-ligand-protein interaction diagram. It was generated for the best poses obtained with orlistat (**A**) and hesperidin (**B**) against LBD-LPT complex systems. Hydrogen bonding interactions are depicted as blue and green dotted arrows in H-bond acceptor/donner pattern, respectively. Besides, the π-π interactions are shown as orange line. The amino acids involved making key interaction with ligand are displayed as purple and green balls.
(TIF)

S1 Table HEDDOCK score of leptin protein docked into leptin binding domain of leptin receptor. (PDF)

S2 Table Surflex score of docked ligands orlistat (ORL) and hesperidin (HES) for Leptin binding domain (LBD) of leptin receptor and leptin protein complex. (PDF)

S3 Table Hydrogen bond analyses from the molecular docking conformation of orlistat and hesperidin in LBD-LPT complex system. (PDF)

S4 Table Comparison between binding free energies of LBD-LPT complex bonded to the inhibitors of orlistat and hesperidin. (PDF)

S5 Table Energy contributions residues in the active site of leptin binding domain bonded to the inhibitors of ORL and HES. (PDF)

S6 Table Comparison between protein-protein interaction energies of ligand-unbonded and ligand bonded LBD-LPT complex system. (PDF)
