# Correction: Hesperidin improves insulin resistance via down-regulation of inflammatory responses: Biochemical analysis and *in silico* validation

**DOI:** 10.1371/journal.pone.0229348

**Published:** 2020-02-12

**Authors:** Kanwal Rehman, Syeda Mehak Munawar, Muhammad Sajid Hamid Akash, Manal Ali Buabeid, Tahir Ali Chohan, Muhammad Tariq, Komal Jabeen, El-Shaimaa A. Arafa

An additional affiliation is missing for the eighth author. El-Shaimaa A. Arafa is also affiliated with Department of Pharmacology and Toxicology, Faculty of Pharmacy, Beni-Suef University, Beni-Suef, Egypt.
