# Supplementary figures and images for: Hesperidin improves insulin resistance via down-regulation of inflammatory responses: Biochemical analysis and in silico validation
Source: PLoS One. 2020 Jan 13;15(1):e0227637. doi: 10.1371/journal.pone.0227637 (PMC6957178; doi:10.1371/journal.pone.0227637)

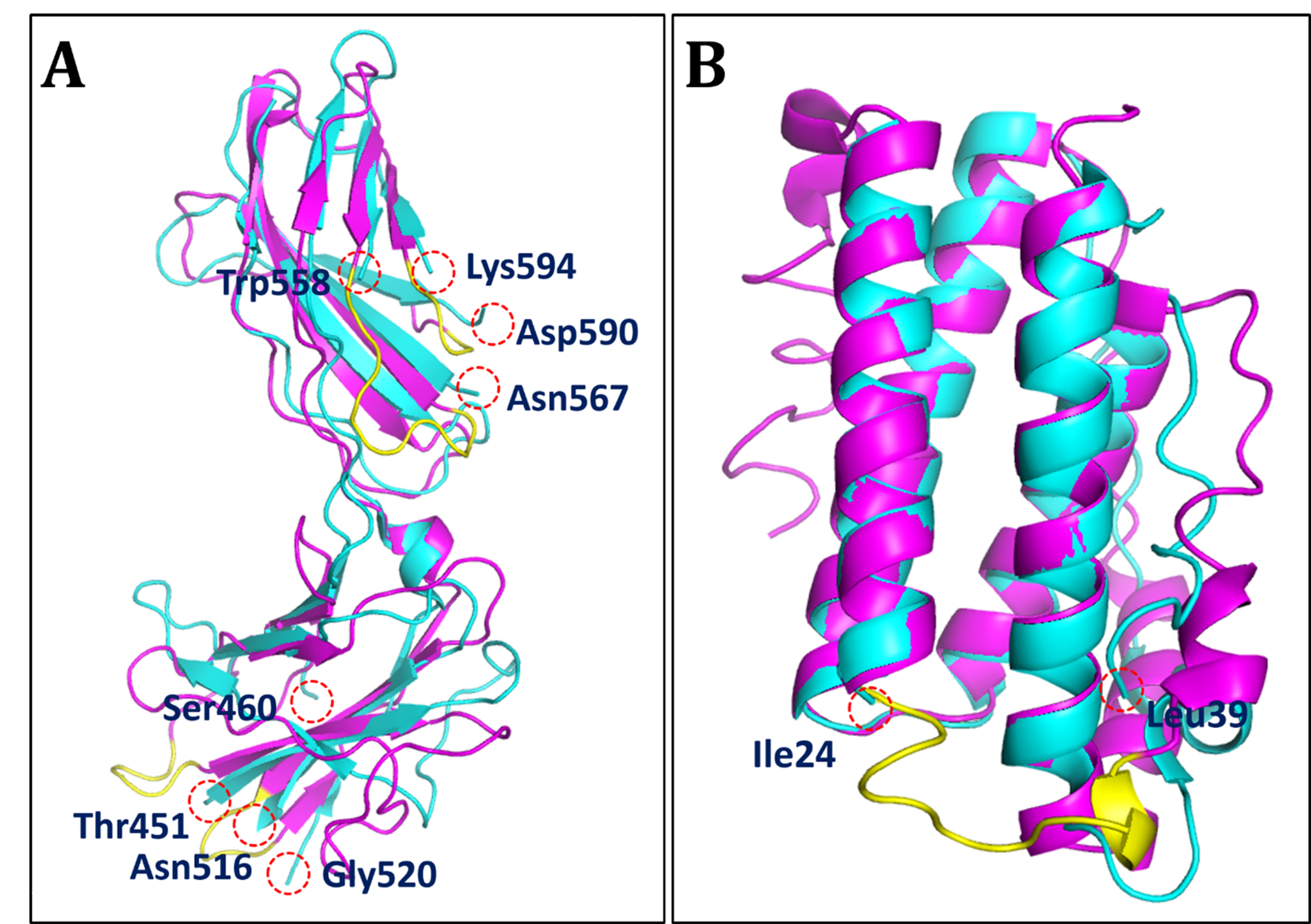

Supplement: S1 Fig — Built by I-TASSER (magenta) superposed over X-ray crystal structure of corresponding proteins (cyan). The loops constructed with molecular modeling are highlighted in yellow color. (TIF) [file pone.0227637.s001.tif]

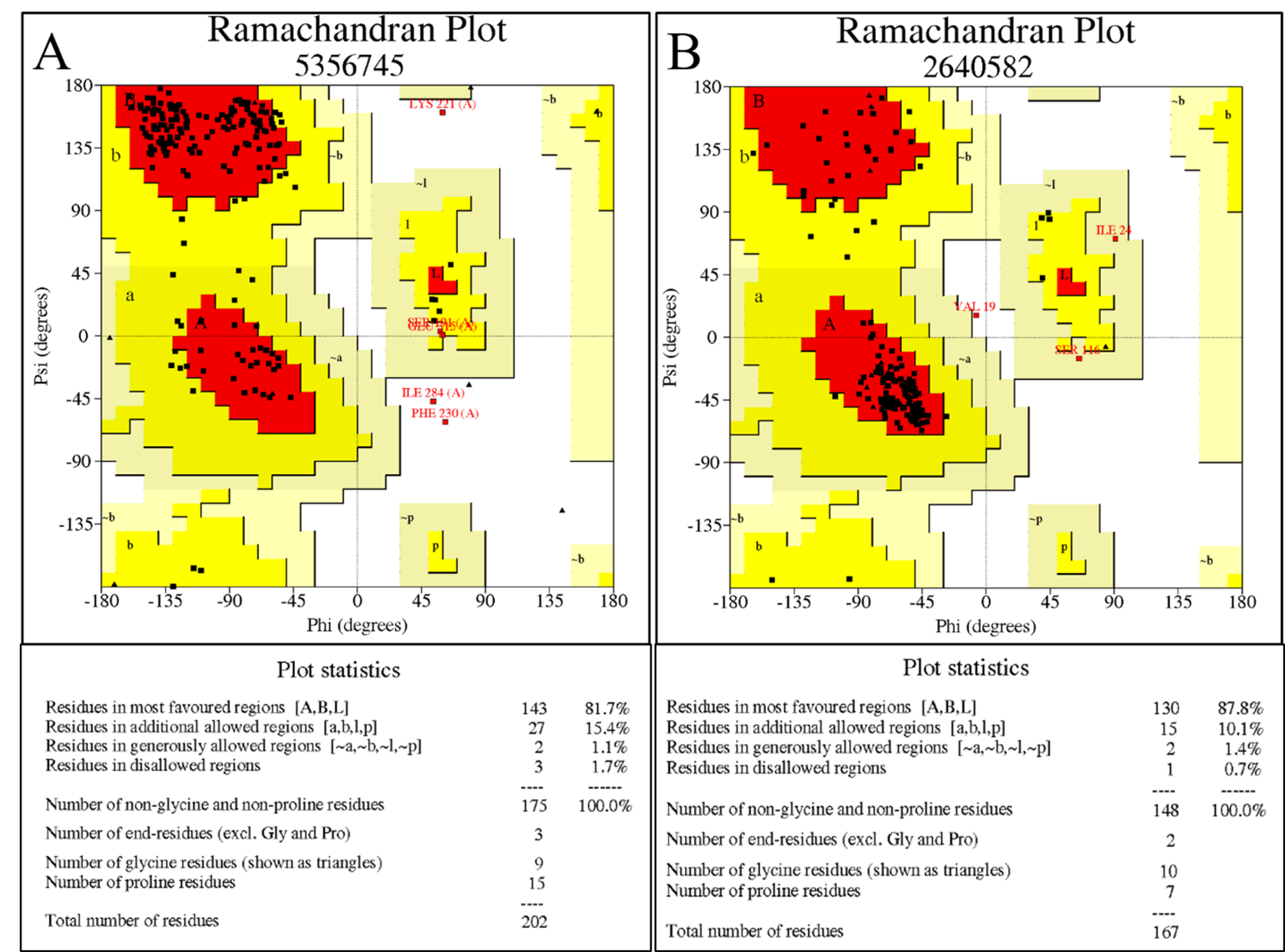

Supplement: S2 Fig — It was performed with RAMPAGE online webserver (A) leptin binding domain of leptin receptor and (E) leptin. (TIF) [file pone.0227637.s002.tif]

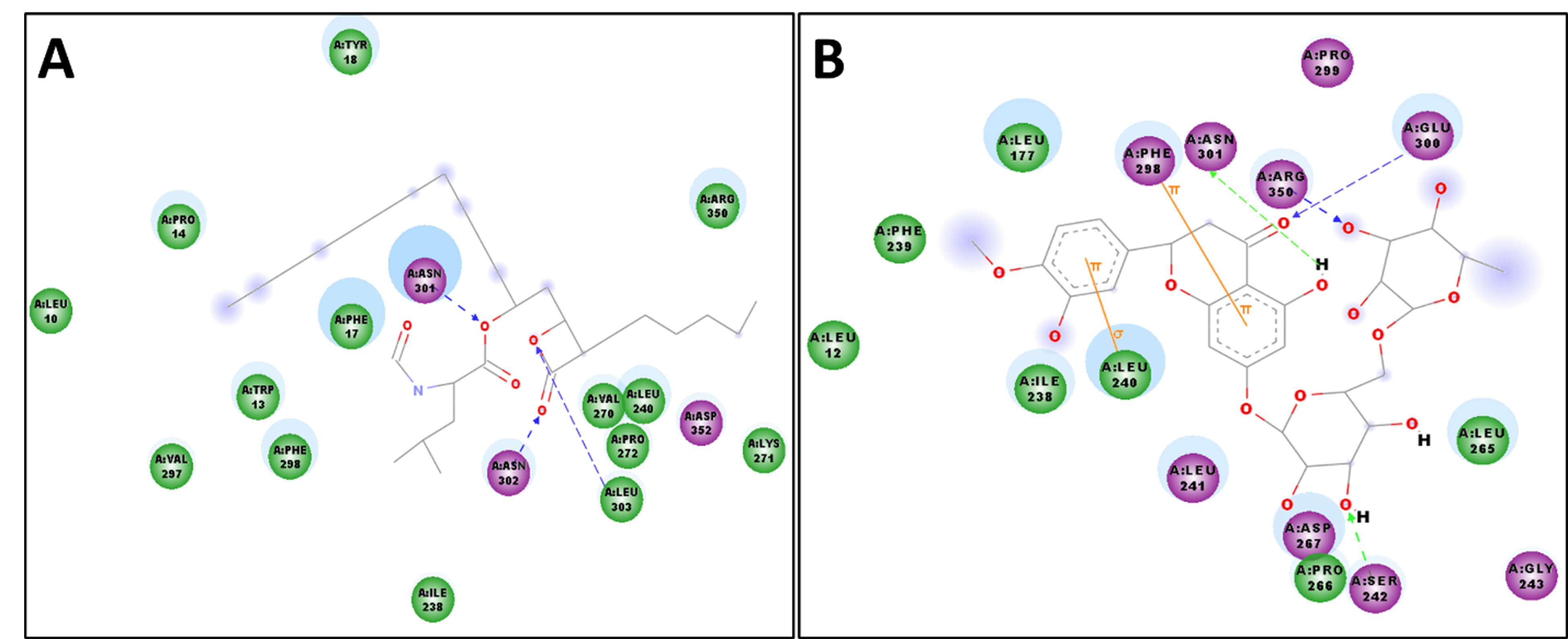

Supplement: S3 Fig — It was generated for the best poses obtained with orlistat (A) and hesperidin (B) against LBD-LPT complex systems. Hydrogen bonding interactions are depicted as blue and green dotted arrows in H-bond acceptor/donner pattern, respectively. Besides, the π-π interactions are shown as orange line. The amino acids involved making key interaction with ligand are displayed as purple and green balls. (TIF) [file pone.0227637.s003.tif]
