# Supplementary material for: Hesperidin improves insulin resistance via down-regulation of inflammatory responses: Biochemical analysis and in silico validation
Source: PLoS One. 2020 Jan 13;15(1):e0227637. doi: 10.1371/journal.pone.0227637 (PMC6957178; doi:10.1371/journal.pone.0227637)
Supplement: S1 Table — (PDF) [file pone.0227637.s004.pdf]

**S1 Table:** HEDDOCK score of leptin protein docked into leptin binding domain of leptin receptor.

| Sr. # | Protein-Protein Docking (HEDDOCK) | Binding affinity scores |
|---|---|---|
| 1 | HEDDOCK score | -152.8 +/- 5.1 |
| 2 | Cluster size | 8 |
| 3 | RMSD from the overall lowest-energy structure | 1.4 +/- 0.5 |
| 4 | Van der Waals energy | -44.0 +/- 1.6 |
| 5 | Electrostatic energy | -26.2 +/- 11.5 |
| 6 | Desolvation energy | -109.3 +/- 4.0 |
| 7 | Restraints violation energy | 57.5 +/- 1.71 |
| 8 | Buried Surface Area | 1273.0 +/- 66.1 |
| 9 | Z-Score | -1.6 |
