# Supplementary material for: Hesperidin improves insulin resistance via down-regulation of inflammatory responses: Biochemical analysis and in silico validation
Source: PLoS One. 2020 Jan 13;15(1):e0227637. doi: 10.1371/journal.pone.0227637 (PMC6957178; doi:10.1371/journal.pone.0227637)
Supplement: S2 Table — (PDF) [file pone.0227637.s005.pdf]

S2 Table. Surflex score of docked ligands **orlistat** (ORL) and **hesperidin** (HES) for Leptin binding domain (LBD) of leptin receptor and leptin protein complex.

Docking complex	CScore [a]	Crash score[b]	Polar score[c]	G score[d]	PMF score[e]	D score[f]	Chem score[g]	Amino acid interaction
ORL-LBD-LPT complex	8.01	-1.11	3.10	-229.548	6.622	-171.798	-5.238	Leu 568, Asn 567, Asn 566,
HES-LBD-LPT complex	10.73	-2.65	3.28	-275.233	40.536	-174.108	-21.996	Glu 565, Asn 566, Ser 507, Asn 567, Arg 615

[a]**CScore** is a consensus scoring which uses multiple types of scoring functions to rank the affinity of ligands, [b]**Crash-**score revealing the inappropriate penetration into the binding site, [c] **Polar** region of the ligand, [d] **G-score** showing hydrogen bonding, complex (ligand-protein), and internal (ligand-ligand) energies, [e] **PMF-score** indicating the Helmholtz free energies of interactions for protein-ligand atom pairs (Potential of Mean Force, PMF), [f] **D-score** for chRe and van der Waals interactions between the protein and the ligand, [g] **Chem-score** points for hydrogen bonding, lipophilic contact, and rotational entropy, along with an intercept term.
