# Supplementary material for: Hesperidin improves insulin resistance via down-regulation of inflammatory responses: Biochemical analysis and in silico validation
Source: PLoS One. 2020 Jan 13;15(1):e0227637. doi: 10.1371/journal.pone.0227637 (PMC6957178; doi:10.1371/journal.pone.0227637)
Supplement: S3 Table — (PDF) [file pone.0227637.s006.pdf]

S3 Table. Hydrogen bond analyses from the molecular docking conformation of orlistat and hesperidin in LBD-LPT complex system.

Protein–ligand systems	H-bond donor	H-bond acceptor	Distance (Å)
ORL-LBD-LPT	Asn566-NH_2	Amino-ester chain-O3	2.792
	Asn567- NH_2	β-lactone-O1	3.060
	Leu568-NH	β-lactone-O2	2.923
HES-LBD-LPT	Ser507-NH	Tetrahydropyran ring (D)-OH	2.994
	Tetrahydropyran ring (D)-OH	Ser507-O	3.244
	Glu565-NH	Tetrahydropyran ring (C)-OH	2.672
	Benzene ring (A)-OH	Asn566-O	2.871
	Asn567-NH_2	Tetrahydropyran ring (E)-OH	3.213
	Arg615-NH_2	Tetrahydropyran ring (E)-OH	2.943
