# Supplementary material for: Hesperidin improves insulin resistance via down-regulation of inflammatory responses: Biochemical analysis and in silico validation
Source: PLoS One. 2020 Jan 13;15(1):e0227637. doi: 10.1371/journal.pone.0227637 (PMC6957178; doi:10.1371/journal.pone.0227637)
Supplement: S4 Table — (PDF) [file pone.0227637.s007.pdf]

S4 Table. Comparison between binding free energies of LBD-LPT complex bonded to the inhibitors of orlistat and hesperidin.

Energy components	ORL-LBD-LPT	HES-LBD-LPT
ΔE_{vdW} [a]	-46.86	-60.11
ΔE_{ele} [a]	-12.82	-70.87
$\Delta G_{nonpol, sol}$ [a]	-6.65	-7.64
$\Delta G_{ele, sol (PB)}$ [a]	34.32	102.18
$\Delta G_{ele, sol (GB)}$ [a]	29.74	91.66
$\Delta E_{vdW}+\Delta G_{nonpol,sol}$ [a]	-53.51	-67.76
$\Delta E_{ele}+\Delta G_{ele,sol (PB)}$ [a]	21.50	31.30
$\Delta E_{ele}+\Delta G_{ele,sol (GB)}$ [a]	16.9	20.78
$\Delta G_{pred (PB)}$ [b]	-30.66	-34.51
$\Delta G_{pred (GB)}$ [b]	-36.58	-46.97

[a] All energies are in kcal/mol, ΔH: the enthalpy changes, [a]$\Delta H = \Delta G_{ele} + \Delta G_{vdW} + \Delta G_{nonpol, sol} + \Delta G_{ele, sol}$, [b] ΔG_{pred}: the calculated binding free energy by MMPB(GB)SA method.
