# Supplementary material for: Hesperidin improves insulin resistance via down-regulation of inflammatory responses: Biochemical analysis and in silico validation
Source: PLoS One. 2020 Jan 13;15(1):e0227637. doi: 10.1371/journal.pone.0227637 (PMC6957178; doi:10.1371/journal.pone.0227637)
Supplement: S5 Table — (PDF) [file pone.0227637.s008.pdf]

S5 Table. Energy contributions residues in the active site of leptin binding domain bonded to the inhibitors of ORL and HES.

Residues around the binding site of LBD[b]	ORL		HES	
	ΔG_v[c]	ΔG_e[d]	ΔG_v[c]	ΔG_e[d]
Pro502	-0.30	0.25	-1.53	0.19
Ile503	-0.06	0.05	-1.88	0.65
Leu505	-0.14	0.02	-2.70	-0.40
Leu506	-0.01	0.04	-3.71	-0.21
Ser507	-0.003	0.014	-1.88	-0.65
Gly508	-0.001	0.008	-1.04	0.45
Leu530	-0.003	-0.004	-1.53	-0.59
Pro531	-0.02	-0.01	-2.34	0.01
Asp532	-0.013	-0.005	-2.08	-0.096
Val535	-0.70	0.16	-0.31	0.01
Pro537	-0.83	0.006	-0.06	0.04
Phe563	-2.83	-0.26	-1.97	0.24
Glu565	-1.42	0.71	-1.47	-1.05
Asn567	-2.33	1.43	-1.10	-0.80
Leu568	-0.51	0.07	-0.05	0.04
Arg615	-1.41	1.00	-0.38	-0.45

[a] All values are in kcal·mol^{-1}, [b] Residues around the binding site of leptin binding domain (LBD) bonded to ORL and HES; [c] $\Delta G_v = \Delta G_{vdW} + \Delta G_{nonpol,sol}$; [d] $\Delta G_e = \Delta G_{ele} + \Delta G_{ele, sol}$
