# Supplementary material for: Hesperidin improves insulin resistance via down-regulation of inflammatory responses: Biochemical analysis and in silico validation
Source: PLoS One. 2020 Jan 13;15(1):e0227637. doi: 10.1371/journal.pone.0227637 (PMC6957178; doi:10.1371/journal.pone.0227637)
Supplement: S6 Table — (PDF) [file pone.0227637.s009.pdf]

S6 Table. Comparison between protein-protein interaction energies of ligand-unbonded and ligand bonded LBD-LPT complex system.

MMPB/GBSA	Protein-Protein binding affinity in ligand bonded & non-bonded systems		
Energy components	LBD-LPT	ORL-LBD-LPT	HES-LBD-LPT
ΔE_{vdW}[a]	-118.11	-132.91	-145.28
ΔE_{ele}[a]	-259.97	-91.60	-96.24
$\Delta G_{nonpol, sol}$[a]	-16.22	-17.94	-19.68
$\Delta G_{ele, sol (PB)}$[a]	323.57	158.78	165.48
$\Delta G_{ele, sol (GB)}$[a]	321.20	162.94	178.45
$\Delta E_{vdW}+\Delta G_{nonpol,sol}$[a]	-134.33	-150.85	-164.68
$\Delta E_{ele}+\Delta G_{ele,sol (PB)}$[a]	63.60	67.78	69.24
$\Delta E_{ele}+\Delta G_{ele,sol (GB)}$[a]	61.23	71.34	82.45
$\Delta G_{pred (PB)}$[b]	-67.14	-80.08	-91.79
$\Delta G_{pred (GB)}$[b]	-72.81	-79.52	-82.75

[a] All energies are in kcal/mol, ΔH: the enthalpy changes, [a]$\Delta H = \Delta G_{ele} + \Delta G_{vdW} + \Delta G_{nonpol, sol} + \Delta G_{ele, sol}$,[b] ΔG_{pred}: the calculated binding free energy by MMPB(GB)SA method.
